# Impact of electric vehicle battery recycling on reducing raw material demand and battery life-cycle carbon emissions in China

**DOI:** 10.1038/s41598-025-86250-1

**Published:** 2025-01-17

**Authors:** Rui Jiang, Chengke Wu, Wei Feng, Kairui You, Jian Liu, Guangmin Zhou, Lujing Liu, Hui-Ming Cheng

**Affiliations:** 1 Institute of Technology for Carbon Neutrality, Shenzhen Institute of Advanced Technology, The Chinese Academy of Sciences, Shenzhen, Guangdong Province People’s Republic of China; 2 School of Material Science and Energy Engineering, Shenzhen University of Advanced Technology, Shenzhen, Guangdong Province People’s Republic of China; 3 Lawrence Berkeley National Laboratory, 94720 Berkeley, CA USA; 4 Energy Research Institute, National Development and Reform Commission (NDRC), Beijing, People’s Republic of China; 5 Tsinghua Shenzhen International Graduate School, Shenzhen, Guangdong Province China; 6 Shenyang National Laboratory for Materials Science, Institute of Metal Research, Chinese Academy of Sciences, Shenyang, Liaoning Province People’s Republic of China; 7 Shenzhen Institute of Advanced Technology, The Chinese Academy of Sciences, Shenzhen, Guangdong Province People’s Republic of China

**Keywords:** Carbon emissions, Battery recycling, Life-cycle emissions, Direct Cathode Recycling, Electric vehicles, Sustainability, Climate-change mitigation

## Abstract

The rapid growth of electric vehicles (EVs) in China challenges raw material demand. This study evaluates the impact of recycling and reusing EV batteries on reducing material demand and carbon emissions. Integrating a national-level vehicle stock turnover model with life-cycle carbon emission assessment, we found that replacing nickel-cobalt-manganese batteries with lithium iron phosphate batteries with battery recycling can reduce lithium, cobalt, and nickel demand between 2021 and 2060 by up to 7.8 million tons (Mt) (67%), 12.4 Mt (96%), and 37.2 Mt (93%), respectively, significantly decreasing reliance on import. Moreover, battery recycling coupled with reuse can reduce carbon emissions by up to 6,532-6,864 Mt (36.0-37.9%), depending on four recycling methods employed. However, this reuse strategy delays battery recycling and risks lithium supply shortage, necessitating trade-offs between carbon reduction and material supply. Future technologies, such as lithium-sulfur and all-solid-state batteries, despite their energy efficiency, might exacerbate lithium shortage, underscoring the crucial need for increased lithium supply.

**Supplementary Information:**

The online version contains supplementary material available at 10.1038/s41598-025-86250-1.

## Introduction

As the world’s largest CO_2_ emitter, China’s goal of achieving carbon neutrality by 2060, with a peak in emissions by 2030, is pivotal^1^. To support this goal, China is in the process of vigorously electrifying. By 2023, China captured 65% of global new electric vehicle (EV) sales, exceeding its 2025 target. Currently, 59% of global EVs are in China^2^. Additionally, vehicle ownership in China remains low, with 226 vehicles per 1000 people, compared to South Korea (485) and Japan (661), indicating significant growth potential^3–5^.

As EV numbers rise, the transportation sector pivots from reliance on traditional fuels to rare materials, elevating concerns from energy to material security^6,7^. Cobalt, lithium, and nickel emerge as critical metals for China’s EV industry due to supply chain risks and economic significance^7^. Previous studies indicate that achieving 40–100% EV penetration by 2050 could significantly increase demand for lithium, cobalt, and nickel, with projected rises of 2909–7513%, 1039–2684%, and 2127–5426%, respectively^8^. Consequently, reducing raw material consumption and adopting a circular economy strategy are viewed as essential solutions to address these global challenges^9–14^. For instance, strategies that focus on high-nickel and low-cobalt content in batteries, as well as battery recycling, are seen as promising approaches to mitigating potential supply risks associated with cobalt in the context of the growing use of ternary lithium-ion batteries^15^. Recently, the Chinese market has shifted towards a preference for lithium iron phosphate (LFP) batteries following the expiration of subsidies. However, retired LFP batteries are often repurposed rather than recycled, due to their low precious metal content^16^, a situation that may evolve. Investigating the risks associated with this preference shift and the end-of-life (EOL) strategies for LFPs is essential for ensuring material and environmental security in China.

With the implementation of the carbon neutrality strategy in China, it is critical to assess the impact of the rapid development of the EV market on carbon emission mitigation as well. While numerous studies have compared the life-cycle emissions of EVs and internal combustion engine vehicles (ICEVs)^17–20^, most have focused on the micro level – typically comparing the life-cycle carbon emissions of a single EV with that of a single ICEV – rather than considering the emissions associated with the entire vehicle fleet. Although Zhang et al. estimated the carbon emission reduction benefits from a system perspective, noting that vehicle electrification significantly contributes to reducing carbon emissions in the global transportation sector^8^, their analysis was limited to the transport phase, rather than the full life cycle. It has been revealed at the micro level that energy- and carbon-intensive processes involved in raw material extraction for battery manufacturing result in high carbon emissions during the production phase^20–22^. Additionally, the carbon footprint of EVs is influenced by the electricity mix, posing significant challenges for coal-dominant countries like China^23,24^. From a system perspective, this situation is further complicated, especially when considering battery recycling and reuse. However, the complete life-cycle carbon emissions of the system (i.e., the vehicle fleet) remain largely unexplored^25^. The potential emission reduction benefits of recycling retired batteries, particularly in the context of second use for peak shaving and valley filling, is still ambiguous. Therefore, this study aims to examine critical material demand and life-cycle carbon emissions in China’s future road transportation electrification from a system perspective, considering the market’s preference for LFP batteries and EOL strategies.

## Methods

This research seamlessly integrated a stock turnover model and a life cycle carbon emission assessment model to comprehensively analyze the trajectory of EVs in the Chinese market. The stock turnover model meticulously projected the future demand for EVs and batteries, considering diverse vehicle types—ranging from private cars to taxis, buses, and trucks. Given the cost advantages, lithium-ion batteries were anticipated to maintain dominance in the market, fueled by advancements in fast-charging technologies and the diminished competitiveness of fuel cell vehicles in the road transportation sector^9,26^. The study focused on two key battery technologies in the Chinese EV market: LFP batteries and nickel-cobalt-manganese ternary (NCM) batteries. The exclusion of explicit discussion on lithium nickel-cobalt-aluminum oxide (NCA) batteries was attributed to their negligible market share in China. Differentiating between private cars, buses, taxis, and trucks, the model accounted for two types of plug-in electric vehicles—plug-in hybrid electric vehicles and battery electric vehicles^27^—paired with two battery groups: LFP and NCM. The model’s predictions encompassed future battery stock and the anticipated number of retired batteries.

**Fig. 1:**
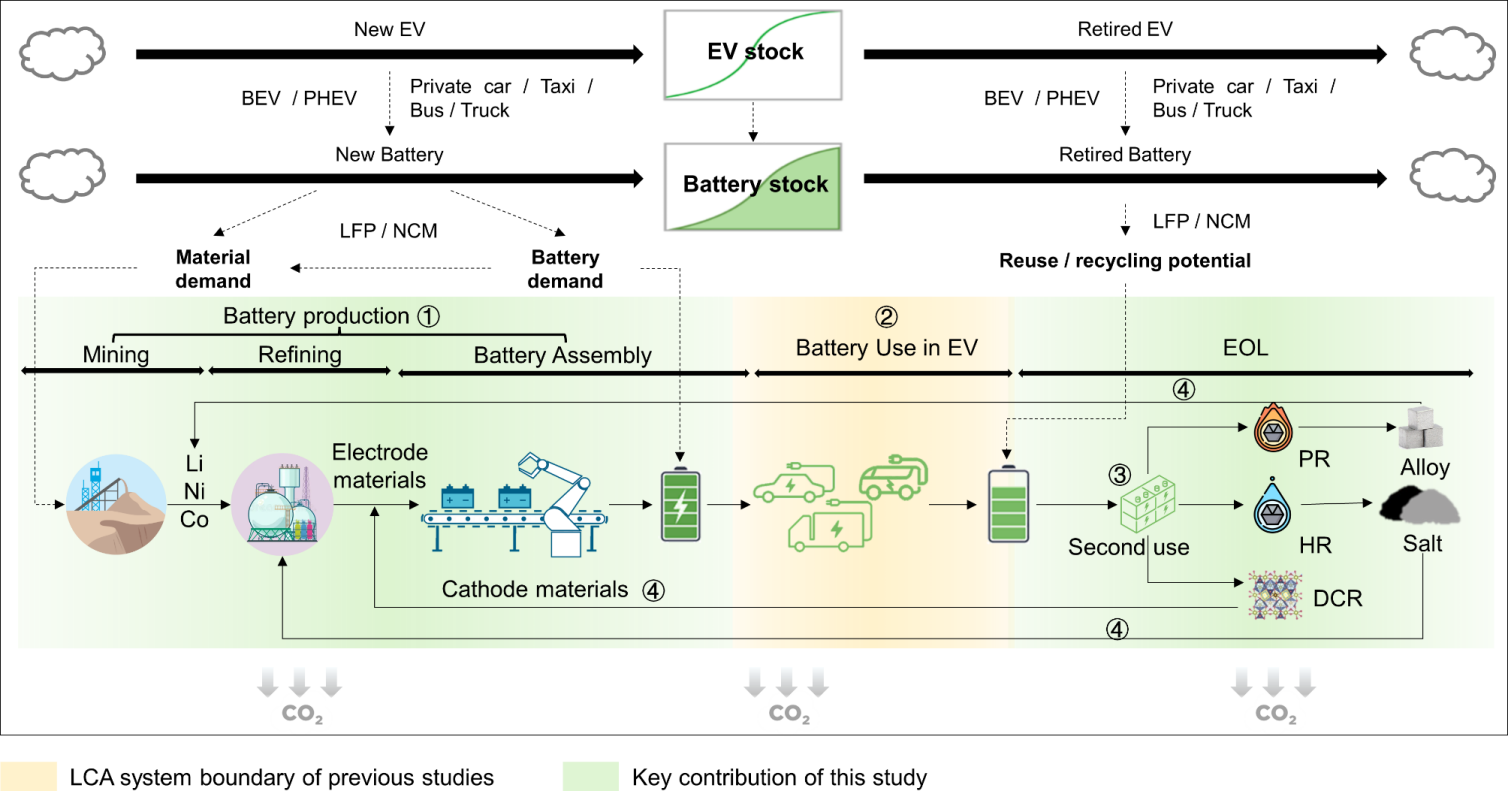
Modeling framework of critical metal demand and life-cycle carbon emissions of EV batteries. This study examines the critical metal demand and life-cycle carbon emissions of EV batteries for various vehicle types, including private cars, taxis, buses, and light, medium, and heavy trucks. The analysis considers two battery technologies: LFP and NCM. The life-cycle carbon emissions of EV batteries are investigated across key phases: battery production (encompassing mining, refining, and assembly), initial battery use in EVs, and EOL stages, involving second use, recycling, and remanufacturing. Additionally, three recycling methods — pyrometallurgical (PR), hydrometallurgical (HR), and direct cathode recycling (DCR) — are compared to assess their environmental impact. BEV – battery electric vehicle; PHEV – plug-in hybrid electric vehicle; Li – lithium; Ni – nickel; Co – cobalt; LCA – life-cycle assessment.

Building on these insights, a comprehensive life cycle carbon emission assessment model calculated carbon emissions throughout the battery life cycle. This included carbon emissions from battery production, incorporating raw material extraction, cathode production, and battery assembly, as well as carbon emissions from battery use in EVs. The assessment extended to EOL processes, including second use, battery recycling, and remanufacturing, visualized in Fig. 1.

### Metal demand projections

In the stock turnover model, the projection of vehicle stocks served as the foundation. Private car, bus, and taxi stocks were determined by multiplying population and ownership per 1000 people, utilizing a dataset from previous publications^28^ deemed more accurate than alternatives^29^. This dataset originates from projections of China’s provincial and gridded population under shared socioeconomic pathways from 2010 to 2100. These projections were generated using a recursive multidimensional approach and validated against historical and contemporary statistical data, including the *China Population and Employment Statistical Yearbooks* and other demographic datasets. Private car ownership was assumed to reach saturation by 2050 at 500 per 1000 people^30^, following a logistic function for growth. Other vehicle types were presumed to grow at historical rates. Truck stocks were computed based on the elasticity coefficient of truck stock growth to GDP growth^31,32^.

The retirement of vehicles was modeled using a Weibull distribution, a widely adopted approach in this context^33^. The basic equation is as follows.

1 $$\:f\left(t\right)=1-\text{e}\text{x}\text{p}\left[{-\left(\frac{t}{\alpha\:}\right)}^{\beta\:}\right]$$

where *f(t)* represented the probability of EV batteries retiring in the *t*th serving year. The shape parameter β, determining the probability density function’s shape, was set at 3.5^34^. The range parameter α was equivalent to the average lifetime of the products studied. For EVs in this study, the baseline scenario assumed an average battery lifetime of 8 years, while other scenarios assumed 10 years^35,36^, consistent with the expected lifetime of the EV batteries^37^. Traditional internal combustion engine private cars, buses, taxis, and trucks had average lifetime of 15 years, 13 years, 8 years, and 15 years, respectively, as per China’s vehicle retirement regulation.

Vehicle demand, based on stocks and retirement, determined EV demand through the multiplication of vehicle demand and EV penetration rate. Supplementary Table 1 outlines key assumptions regarding the EV penetration rate among new vehicles from 2020 to 2060. Additionally, Supplementary Tables 2–4 detail the allocation of battery types (LFP and NCM) to vehicle types from 2020 to 2060 across the four scenarios. EV stock levels were then obtained, and battery demand, stock, and retired batteries were calculated using different battery capacity data for the four EV types. These results were subsequently utilized to assess critical metal demand based on battery composition^38^. To evaluate the potential risk of critical material supply shortages, we calculated the difference between material demand and supply, including both raw and recycled materials. To ensure production availability and account for limited mine reserves in China^39^, we conservatively assumed that raw material supply would remain at the 2020 production levels.

In addition, assuming that future batteries technologies such as lithium-sulfur batteries (LSBs) and all-solid-state batteries (ASSBs) may reduce lithium demand, a comparison of lithium demand among lithium-ion batteries, LSBs, and ASSBs was conducted. As such, data for lithium-ion batteries were derived from existing literature^38^, while estimates for LSBs and ASSBs were based on material inventories compiled from a published study^40^.

### Life-cycle carbon emissions calculation

Annual life-cycle carbon emissions were calculated based on projected yearly battery demand, stocks, and retired batteries. Cumulative carbon emissions were then obtained by summing the annual carbon emissions over time. Equations (2)-(8) outline the calculation process:

2 $$\:{E}_{total,t}={E}_{p,t}+{E}_{u,t}+{E}_{eol,t}$$

where *E*_*total, t*_ represented total carbon emissions in year t (from 2021 to 2060), and *E*_*p, t*_, *E*_*u, t*_, and *E*_*eol, t*_ corresponded to carbon emissions from the production, use, and EOL phases in year *t*, respectively. In this study, all reported carbon emissions were expressed as greenhouse emissions in CO_2_-equivalent (CO_2_-eq). We focused specifically on the life cycle of EV batteries rather than entire vehicles, as emissions from vehicle production or EOL had minimal impact on the choice of battery chemistry. However, the carbon emission reduction benefits during the use phase for the transport sector were calculated separately, providing results that are comparable to similar studies and offering a more comprehensive view of the life-cycle carbon emissions of EV batteries. While second-use batteries can reduce the demand for new batteries, these benefits were not included in this study to maintain clarity in the system boundaries and focus on the direct environmental impacts of the battery’s lifecycle. Including indirect benefits, such as reduced new battery production, introduces uncertainties related to technological developments in energy storage, market dynamics, and future demand projections for stationary storage, which fall outside the scope of this study. Additionally, allocating these benefits across lifecycle stages would complicate the result interpretation and may not align with the methodology of similar studies.

3 $$\:{E}_{p,t}={E}_{p1}\times\:{D}_{t}$$

where *E*_*p1*_ represented the carbon emissions per kWh of battery production, and *D*_*t*_ was the total demand for new batteries in year *t*.

4 $$\:{E}_{u,t}=\sum\:_{j=1}^{5}{E}_{u1,t,j}\times\:{S}_{j,t}=\sum\:_{j=1}^{5}{EF}_{t}\times\:{VMT}_{j,1}\times\:{EE}_{j,t}\times\:{S}_{j,t}$$

where *E*_*u1,t, j*_ represented the carbon emissions of a single vehicle of type *j* in year *t* (where *j* ranged from 1 to 5, representing car, bus, taxi, light truck, and heavy truck); *S*_*j, t*_ was the vehicle stock of type *j* in year *t*; *EF*_*t*_ was the emission factor per kWh of electricity use in year *t*, determined by the electricity mix; *VMT*_*j, 1*_ represented the vehicle miles travelled by a single vehicle of type *j* (in kilometers); and *EE*_*j, t*_ referred to the energy efficiency of vehicle type *j* in year *t* (in kWh/km). A similar equation was also used to calculate carbon emissions for ICEVs during the use phase, to assess the carbon emission reduction benefits of transitioning to EVs in the transport sector.

5 $$\:{E}_{eol,t}=\:{E}_{2nd,t}+{E}_{re,t}$$

where *E*_*2nd, t*_ and *E*_*re, t*_ referred to carbon emission benefits from second use and from recycling and remanufacturing of retired batteries in year *t*, respectively.

6 $$\:{E}_{2nd,t}={E}_{2nd1,t}\times\:{SB}_{t}\:$$

where *E*_*2nd1,t*_ represented the carbon emission benefits from the second use of one kWh of retired batteries in year *t*, determined by the charging and discharging cycle and depth of discharge, which were assumed to be 365 cycles per year and 75%, respectively, in this study^41^; *SB*_*t*_ was the stock of retired batteries in second use in year *t*, calculated using the stock turnover model described above.

7 $$\:{E}_{re,t}={E}_{re1,t}\times\:{B}_{t}$$

where *E*_*re1,t*_ represented the carbon emission benefits of recycling one kWh of retired batteries for manufacturing in year *t*; *B*_*t*_ was the amount of retired batteries recycled for remanufacturing in year *t*.

8 $$\:{E}_{cumulative,t}={E}_{cumulative,t-1}+{E}_{t}$$

where *E*_*cumulative*_, *t* referred to the cumulative carbon emissions in year *t*, calculated by adding the carbon emissions of year *t* to the cumulative carbon emissions from year *t-1*. This equation applied to both total carbon emissions and emissions from specific life-cycle phases.

Essential emissions data for a single battery life cycle were sourced from published literature. For consistency, all data used in this study originated from the same research team, encompassing battery production emissions data^22^, various electricity mix data^21,22,42–44^ for calculating carbon emissions from battery use in EVs, and battery EOL emissions data^21,45^. Detailed data about manufacturing processes, recycling and remanufacturing processes and allocation methods can be accessed through these studies. The assumed lifetime of second-use retired EV batteries was set at 10 years^46^. Additionally, recycling and remanufacturing benefits of three battery recycling methods were considered in battery EOL, including pyrometallurgy^21^, hydrometallurgy^45^, and two direct cathode recycling methods^45^.

### Scenarios of future battery demand and life-cycle carbon emissions

In this study, four scenarios were established based on different battery technologies to evaluate future critical material demands and life-cycle carbon emissions of EV batteries in China (Supplementary Table 5). Scenario 1 (S1) served as the baseline, reflecting current battery technology with an 8-year average EV battery lifetime, a higher NCM market share than LFP, and medium-nickel technologies dominating NCMs. Scenario 2 (S2) extended the battery lifetime to 10 years based on S1. Scenario 3 (S3) took a more assertive stance, opting for an LFP-dominant path over NCM, building on S2. Scenario 4 delved deeper into a high-nickel oriented path for NCM technology development based on S3.

For each scenario, metal requirements and carbon emissions results were analyzed to depict the situation without EOL strategies. Subsequently, we assessed the impact of different EOL strategies, encompassing the adoption or exclusion of second use and the three battery recycling methods. Second use adoption rates of 0%, 20%, 40%, 60%, 80%, and 100% were considered to assess the impact of varying levels of second use implementation. Comparing different EOL strategies allowed us to understand how the market preference for LFPs in China influenced the outcomes.

## Results

### Critical metal demand without end-of-life strategies

**Fig. 2:**
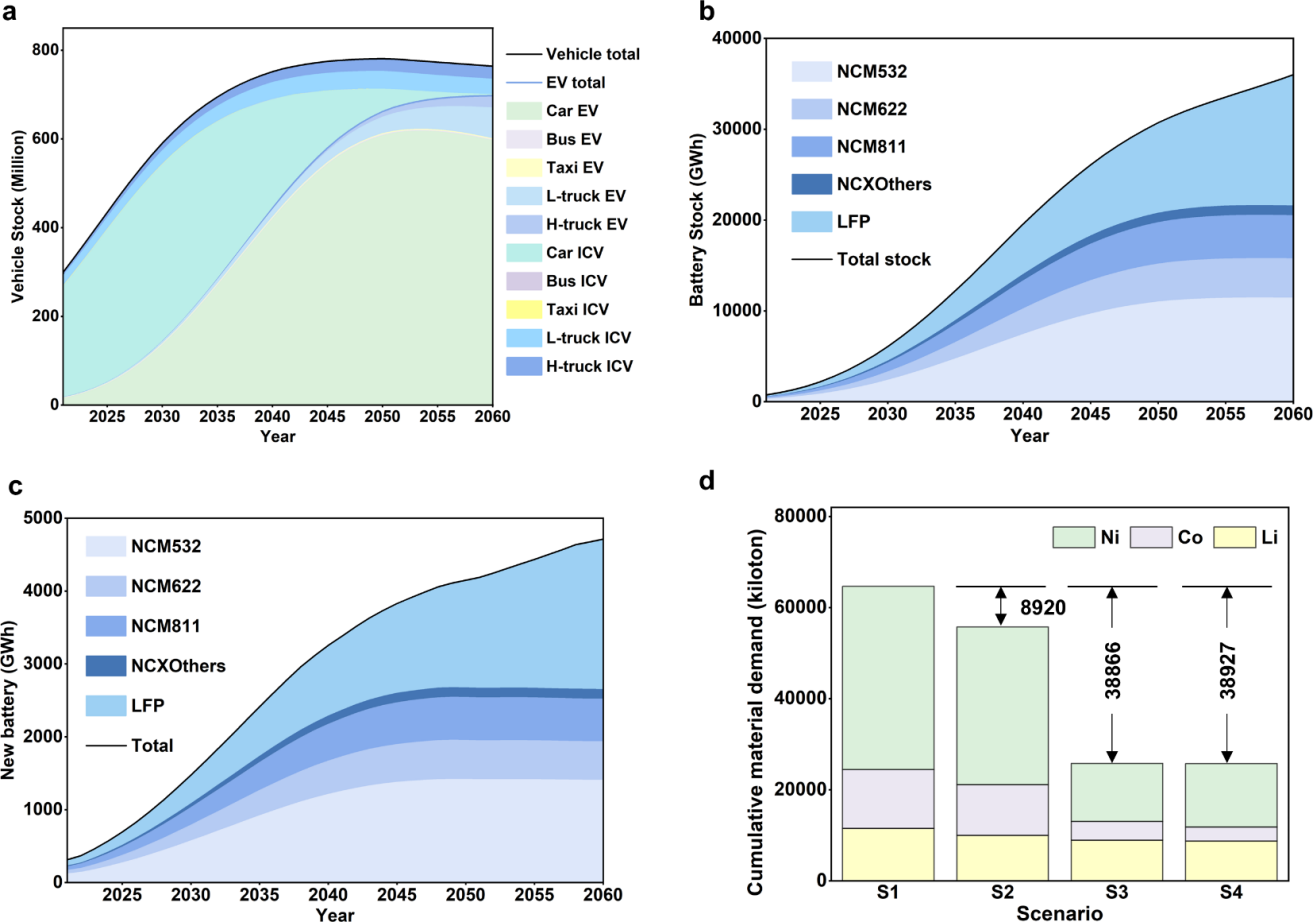
Battery stock, demand, and the corresponding metal demand. (**a**) Annual vehicle stock under the baseline scenario. (**b**) Annual battery stock under the baseline scenario. (**c**) Annual demand for new batteries under the baseline scenario. (**d**) Cumulative material demand under various scenarios. S1 – baseline scenario; S2 – S1 + extending battery lifetime to ten years; S3 – S2 + LFP-dominant approach; S4 – S3 + high-nickel batteries.

In S1, we project the stock of EVs in China to increase to 143 million by 2030 and 698 million by 2060, while internal combustion engine vehicles decline. Electric cars will constitute 95.1% and 85.5% of the EV total, respectively (see Fig. 2a). This corresponds to 6,078 GWh of batteries in use by 2030, with NCM 532 being the most prevalent at 39.1%. By 2060, the total battery capacity will reach 36,000 GWh, with LFP batteries leading at 40.1% and NCM 532 declining to 31.7% (see Fig. 2b). Figure 2c illustrates the annual demand for new batteries under S1, projecting a more than tenfold increase by 2030 and a nearly fortyfold increase by 2060. Results for other scenarios are detailed in Supplementary Fig. 1.

To meet this demand, a cumulative 11.5, 12.9, and 40.2 million tons of lithium, cobalt, and nickel production are required under current battery technology (see Fig. 2d). Extending battery lifetime from 8 to 10 years (S2) reduces demand by 13.1% for lithium and 13.9% for cobalt and nickel. Adopting an LFP-dominant strategy based on S2 (S3) offers a further 9.2% and 54.4% reduction, respectively. Transitioning to high-nickel batteries on the basis of S3 (S4) reduces lithium and cobalt demand by 1.6% and 8.0%, respectively, but requires 2.9% more nickel than S3. Overall, S3 and S4 reduce metal demand by 60.1% and 60.2% compared to the baseline, respectively.

Supplementary Fig. 2 delineates the annual demand trends for lithium, cobalt, and nickel across various scenarios. In all four scenarios, lithium demand consistently ascends until 2060. The highest demand, recorded in S1 for 2060, reaches 475 kilotons. In contrast, cobalt and nickel demand increases until around 2050 in S1 and S2, then stabilizes. In S3 and S4, where LFP-dominant strategies are adopted, their peak demand occurs around 2040 and then gradually declines to near-zero by 2060.

### Raw material demand reduction potential across various end-of-life strategies

**Fig. 3:**
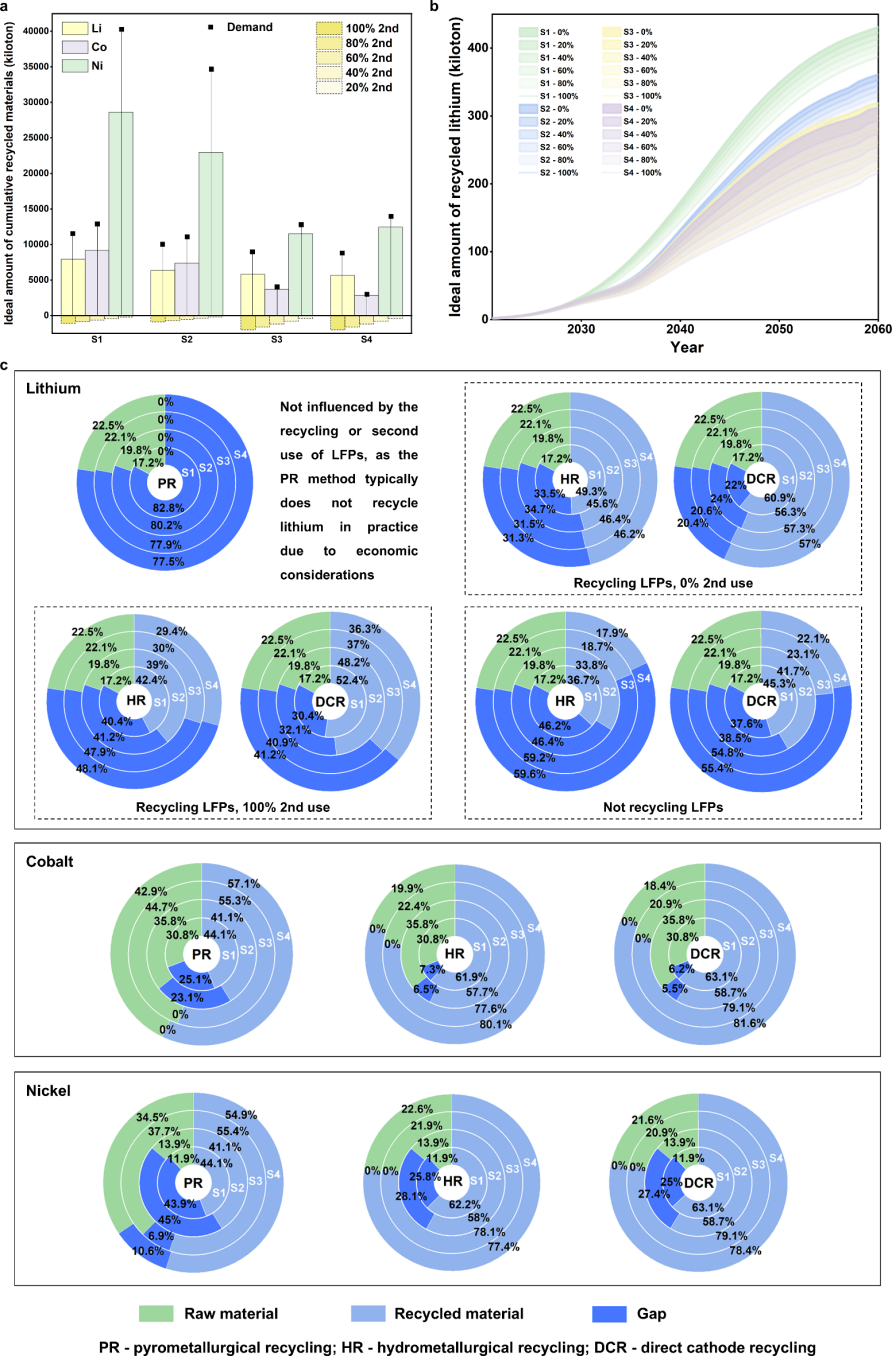
Recycling potential under diverse scenarios. a Cumulative recycled lithium, cobalt, and nickel compared to their cumulative demand under various scenarios (dashed rectangles denote the reduced lithium recycled from second use of retired LFPs). **b** Detailed information regarding the amount of recycled lithium under different second-use ratios and scenarios. **c** Contribution of cumulative recycled metal to cumulative metal demand and the supply gap using different recycling methods. Cumulative raw material supply is calculated based on China’s 2020 production levels. 0% and 100% represent scenarios where retired LFPs are subjected to 0% and 100% second use, respectively.

In an ideal scenario where all metals are 100% recycled, cumulative recycled cobalt and nickel could reduce raw material production by at least 66.3% (S2) (see Fig. 3a). In S3 and S4, this contribution exceeds 90%, highlighting significant potential to mitigate risks of critical material supply shortages given China’s reliance on import. Note that NCM batteries are not considered for second use due to China’s safety regulations, as they are more prone to thermal runaway and overheating compared to other chemistries, such as LFP. The most significant contribution of lithium recycling to reducing virgin lithium demand occurs under the baseline scenario, with a peak reduction of 68.7% when second use is not implemented. This is because while alternative scenarios reduce lithium demand relative to the baseline, extending battery lifetime also delays recycling. The contribution slightly decreases to 64 − 65% in other scenarios. It is important to note that the second use of retired LFPs can significantly decrease the amount of recycled lithium under S3 and S4 by more than 22%. This effect is limited in S1 and S2 due to the relatively low proportion of LFPs (see Fig. 3a and b).

Based on real-life collection and recycling rates, the recycled amounts of lithium, cobalt, and nickel can be reduced by 11.1–100% (with the PR recycling method recycling no lithium in practice due to economic considerations), 12.1–38.5%, and 12.0–38.4%, respectively, compared to the ideal amounts, depending on the recycling method. The real-life collection rate is defined as the proportion of retired batteries actually collected and diverted from the waste stream, divided by the total amount generated. The real-life recycling rate is the mass of material actually recycled divided by the total generated, expressed as a percentage^47^. Detailed data on the collection and recycling rates are provided in Supplementary Tables 6–7. Figure 3c illustrates the contribution of recycled materials to fulfilling lithium, cobalt, and nickel demand at a real-life collection and recycling rate. In general, DCR makes the most significant contribution to reducing raw material demand due to its highest recycling rate. When comparing different scenarios, LFP-dominant ones (S3 and S4) exhibit smaller material supply shortages when both NCM and LFP batteries are recycled without second use. This is primarily due to their relatively low material demand. However, even in the best-case scenarios (recycling LFPs with no second use under S4), lithium demand remains unmet by at least 20.4%. Supplementary Fig. 3a presents detailed results from a sensitivity analysis, highlighting the need for over a twofold increase in annual lithium production from 2020 level. It is worth noting that S3 and S4 are highly sensitive to the second use of retired LFPs, which can reduce recycled lithium availability and increase lithium shortages for EVs by 20% (see Fig. 3c and Supplementary Fig. 3b). This high sensitivity is attributed to the significant proportion of LFPs in these two scenarios. Additionally, not recycling LFPs exacerbates the supply shortages in all scenarios (see Fig. 3c and Supplementary Fig. 4).

**Fig. 4:**
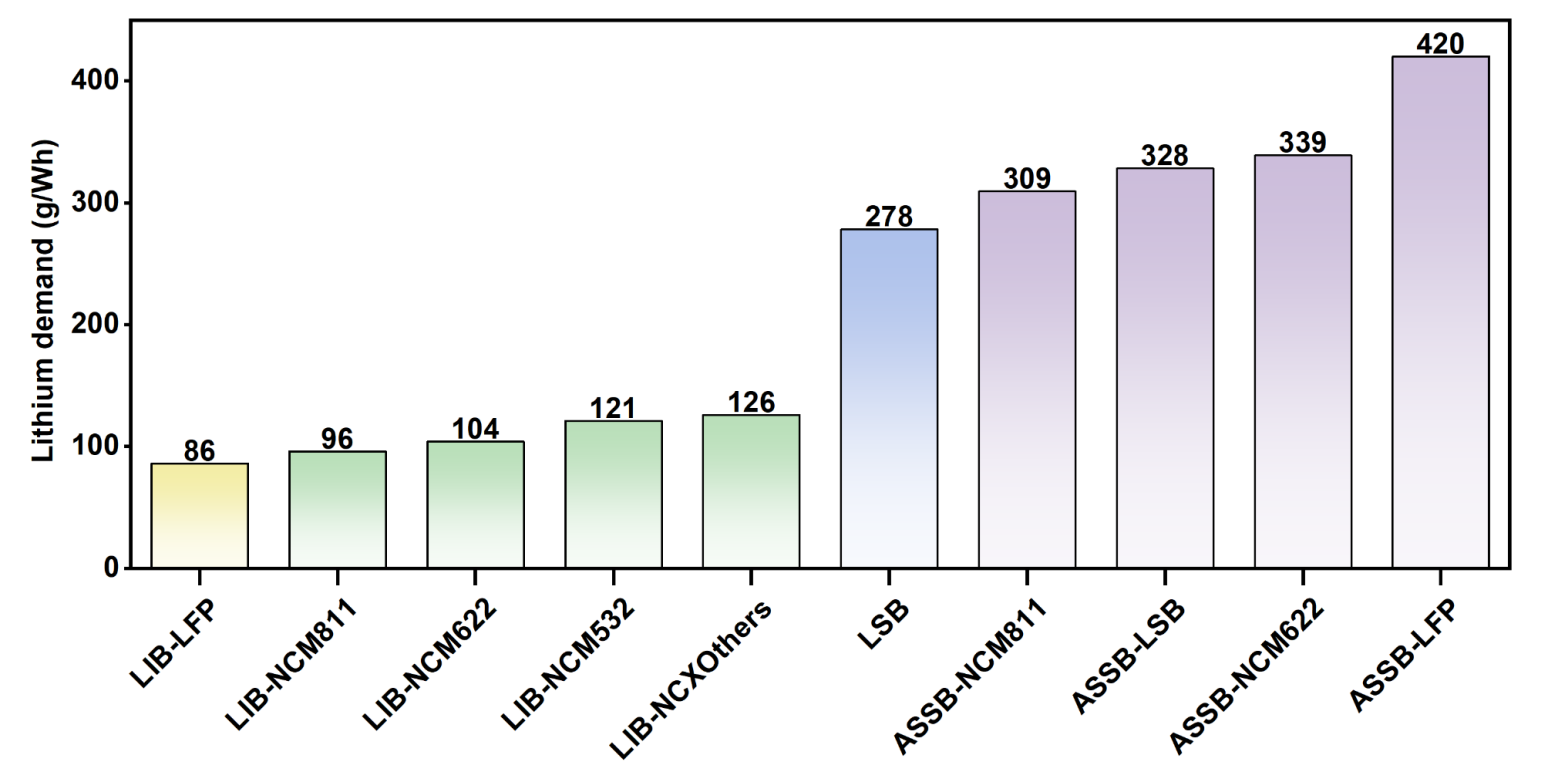
Lithium demands different battery technologies. LIB – Lithium-ion battery. LSB – Lithium-sulfur battery. ASSB – All-solid-state battery.

Hence, we extend our analysis to investigate the lithium demand of various promising future battery technologies, encompassing lithium-sulfur and all-solid-state batteries utilizing LFP, NCM, and sulfur as positive active materials. Remarkably, all these technologies exhibit substantially higher lithium demand compared to traditional LFP and NCM batteries. Figure 4 presents a comparison of lithium demand per watt-hour of battery capacity across various battery technologies. The analysis reveals that emerging technologies, notably lithium-sulfur batteries and all-solid-state batteries, are projected to at least double lithium requirements per watt-hour of battery production. This surge is primarily attributed to the extensive use of lithium metal as an anode material.

In scenarios S3 and S4, cobalt demand can be met through any of the three recycling methods, whereas nickel demand can be addressed specifically by the HR or DCR approaches (Fig. 3c). However, this assumes all produced metal is used for EV production, which is not always the case. When the available metal for EV production drops below 81% of the 2020 production level, nickel demand can only be satisfied under S3 with the DCR method. It becomes unmet when the proportion falls to 72.8%. Cobalt supply is more robust, as China refines 70% of global cobalt^48^. Under S3, a shortfall occurs only when cobalt supply for EVs falls below 29.7% of the 2020 production level using the HR method or 28.2% with the DCR method. For detailed results, refer to Supplementary Fig. 3.

### Life-cycle carbon emissions under different battery technologies and end-of-life strategies

**Fig. 5:**
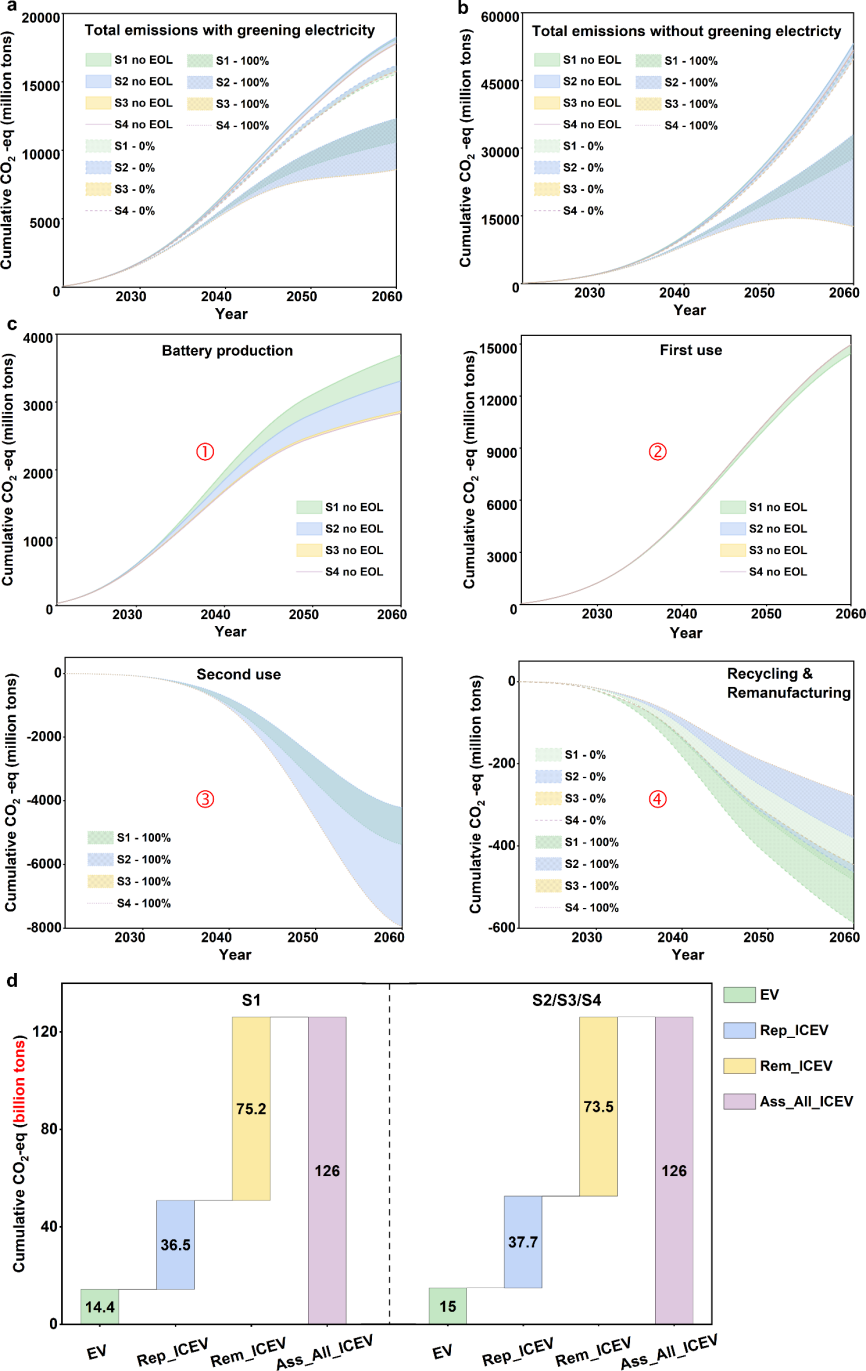
Life-cycle carbon emissions of EV batteries in the studied system. (**a**) Life-cycle carbon emissions considering future electricity mix change (using the DCR method). (**b**) Life-cycle carbon emissions without considering future electricity mix change (using the DCR method). (**c**) Life-cycle carbon emissions of EV batteries. ①battery production, encompassing raw material extraction, cathode production and assembly. ②electricity use in transportation. ③second use of retired LFPs. ④remanufacturing benefits of batteries with recycled lithium, cobalt, and nickel. (**d**) Reduced carbon emissions through the replacement of ICEVs with EVs. Rep_ICEV – replaced ICEV with the EVs in the system; Rem_ICEV – ICEVs remained in the system; Ass_All_ICEV – assuming all vehicles in the system are ICEVs. 0% and 100% represent scenarios where retired LFPs are subjected to 0% and 100% second use, respectively.

Figure 5 displays the cumulative life-cycle carbon emissions of batteries in the system, using the DCR method for illustration. Without EOL processing, a total of 18,134 million tons of carbon emissions are projected under S1 (see Fig. 5a). For S2, S3, and S4, the results are 18,271, 17,825, and 17,794 million tons, respectively. Notably, EOL strategies significantly reduce life-cycle carbon emissions, especially with second use (22.6 − 47.3% reduction for DCR, varying by scenarios). Furthermore, S3 and S4 consistently yield the lowest carbon emissions, regardless of second use adoption and recycling strategies, even in cases where retired LFPs are not recycled (see Supplementary Fig. 5). This indicates the significant carbon emission benefits of transitioning from NCMs to LFPs. Unexpectedly, in S2, significantly higher carbon emissions are observed than S1 when retired LFPs are entirely reused. This is primarily due to the reduced number of retired batteries resulting from the prolonged battery lifetime.

Figure 5b displays potential carbon emission trends with an unchanged electricity mix. The results reveal that a cumulative increase of 33,846 − 34,955 million tons (190-193%) in carbon emissions could occur without EOL. With the adoption of second use, this difference can be reduced to 3,009–20,599 million tons, varying by scenarios and recycling methods. The substantial carbon emission reduction is attributed to avoided electricity generation through reusing retired LFPs for peak shaving and valley filling. Furthermore, the carbon emission reduction potential from recycling without second use is limited, reaching a maximum of 4.7% (S1, DCR-C) (see Supplementary Fig. 5). The carbon benefits of recycling are modest, which contrasts with findings from micro-level LCA studies (e.g., for a single EV). This is primarily due to the relatively small share of carbon emissions from battery production compared to those from battery use within the broader system (e.g., for the EV fleet), as the production of new batteries is much lower than the existing battery stock. With 100% second use adoption, the maximum potential reduction could increase to 75.7% (S4, DCR-C).

Figure 5c illustrates carbon emissions across various phases of the battery life cycle. As the demand for batteries rises, carbon emissions from production exhibit rapid accumulation until approximately 2048. Subsequently, the pace of carbon emission growth decelerates owing to a stabilized demand for batteries and the transition toward greener electricity sources (see Fig. 5c①). NCM battery production generates more carbon emissions than LFP batteries due to energy- and emission-intensive cobalt and nickel mining and processing^45^, resulting in higher carbon emissions for scenarios S1 and S2.

Carbon emissions resulting from the utilization of batteries in EVs exhibit a consistently high and relatively steady cumulative rate since 2030 (see Fig. 5c②). The S1 scenario generates the least electricity-use carbon emissions due to its lower EV stock, totaling 14,443 million tons. However, this also implies the smallest carbon emission reduction from replacing ICEVs with EVs, at 36,504 million tons for S1 (a 72% reduction). The other three scenarios achieve a reduction of 37,736 million tons, significantly higher than the total life-cycle carbon emissions of the batteries (see Fig. 5a and d). Additionally, scenarios S2, S3 and S4 save an extra 1,753 million tons of carbon emissions relative to S1 owing to a lower remaining share of ICEV in the stock (see Fig. 5d). Excluding EOL strategies, the use phase accounts for more than 80% of the cumulative carbon emissions.

With EOL strategies considered, the benefits of second use for carbon emission reduction increase rapidly with its stock until around 2050, after which they slow down due to diminished electricity generation emissions (see Fig. 5c③). Notably, scenarios S3 and S4 offer significantly higher carbon emission reduction potential than S1 and S2, owing to their greater proportion of LFPs, which can be repurposed for second use after retirement. Moreover, the advantages of recycling and remanufacturing remain inferior to those of second use (see Fig. 5c④). Given the higher carbon emissions associated with NCM production compared to LFPs, S1 yields the highest recycling and remanufacturing benefits by reducing raw material use. Additionally, S3 and S4 show greater sensitivity to second-use strategies due to their higher proportion of LFPs. Supplementary Fig. 5 compares different recycling methods. Without second use, recycling retired batteries results in an average reduction of total carbon emissions by 3.4%, 2.8%, 2.1%, and 0.8% with DCR-C, DCR-B, HR, and PR, respectively. In contrast, with 100% second use, the maximum potential carbon emission reduction associated with these figures escalates to 37.9%, 37.5%, 37.1%, and 36.0%, respectively. As previously noted, the limited carbon emission reduction benefits of recycling arise mainly from the relatively small contribution of battery production emissions compared to those from battery use within the broader system.

## Life-cycle carbon emissions per kWh of battery stock in EVs

**Fig. 6:**
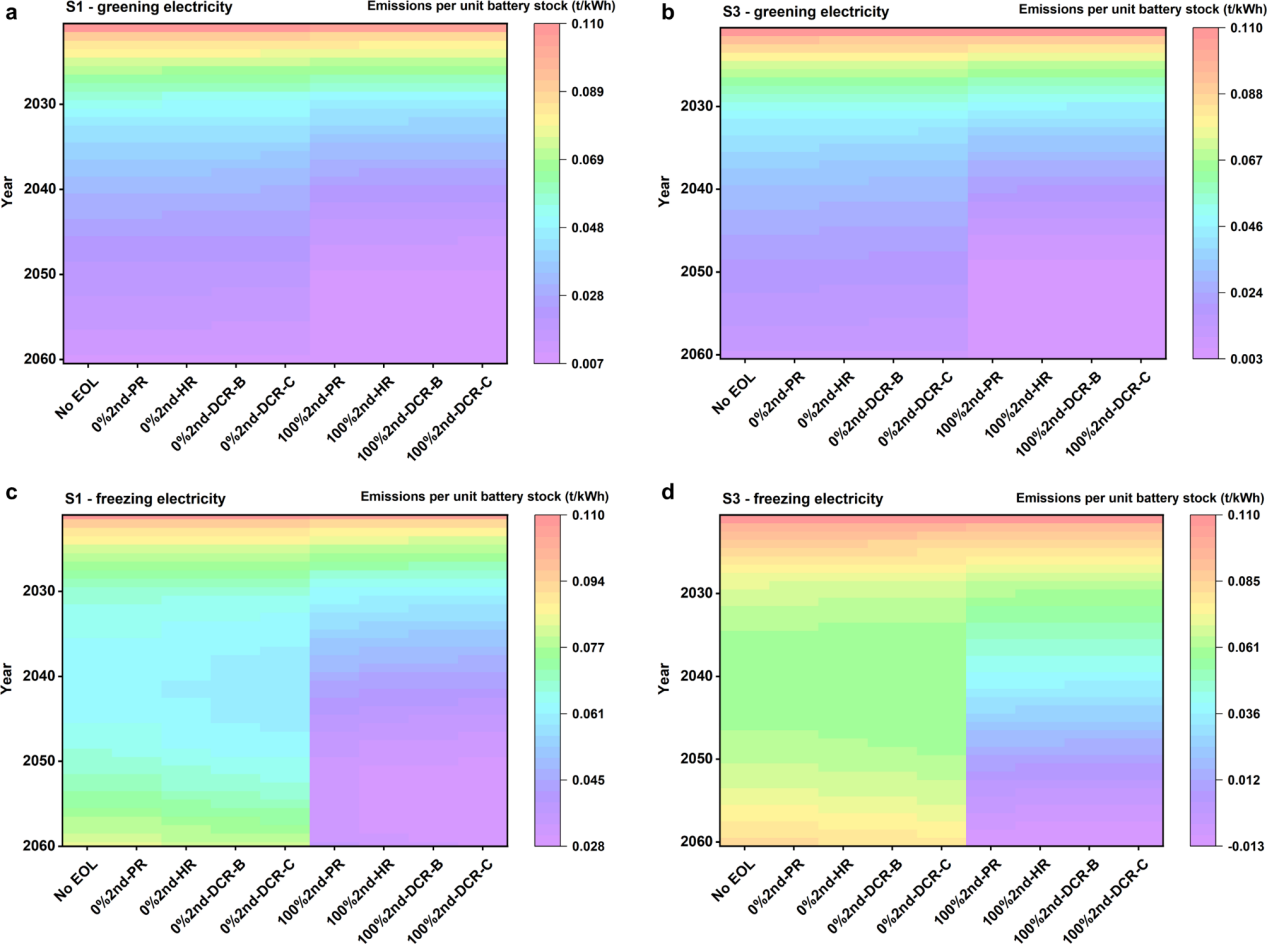
Carbon emissions per kWh of battery stock in the first use. (a) S1 with a transition to a greener electricity mix. (**b**) S3 with a transition to a greener electricity mix. (**c**) S1 without considering the transition to a greener electricity mix. (**d**) S3 without considering the transition to a greener electricity mix. Only results for S1 and S3 are presented, as S1 and S2 exhibit a similar pattern, and S3 and S4 display comparable trends.

Given the primary application of the studied battery system in transportation, we establish the functional unit as carbon emissions per unit kWh of battery stock utilized in EVs. Our findings indicate a similar pattern between S1 and S2, whereas S3 and S4 exhibit comparable trends. When accounting for the transition to a greener electricity mix, unit carbon emissions consistently decrease across all scenarios (see Fig. 6a and b). Particularly noteworthy is the consistent trend of lower carbon emissions in S3 and S4 than in S1 and S2, irrespective of the adoption of second use. Additionally, S3 and S4 exhibit more substantial benefits derived from second use.

When the electricity mix remains constant, unit carbon emissions experience a rapid decline from 2021 to 2030, followed by a deceleration until 2040. Subsequently, there is an upward trajectory (refer to Fig. 6c and d), primarily attributed to reduced recycling benefits due to declining new battery demand and a continuous rise in carbon emissions from EV utilization. However, with the adoption of 100% second use, unit carbon emissions consistently decrease. Remarkably, owing to the substantial advantages of second use, unit carbon emissions for S3 and S4 even turn negative starting in 2054. It is evident that the carbon emission reduction benefits from second use far exceed those of the greener electricity mix scenarios.

### Trade-offs between life-cycle carbon emissions and recycled materials

**Fig. 7:**
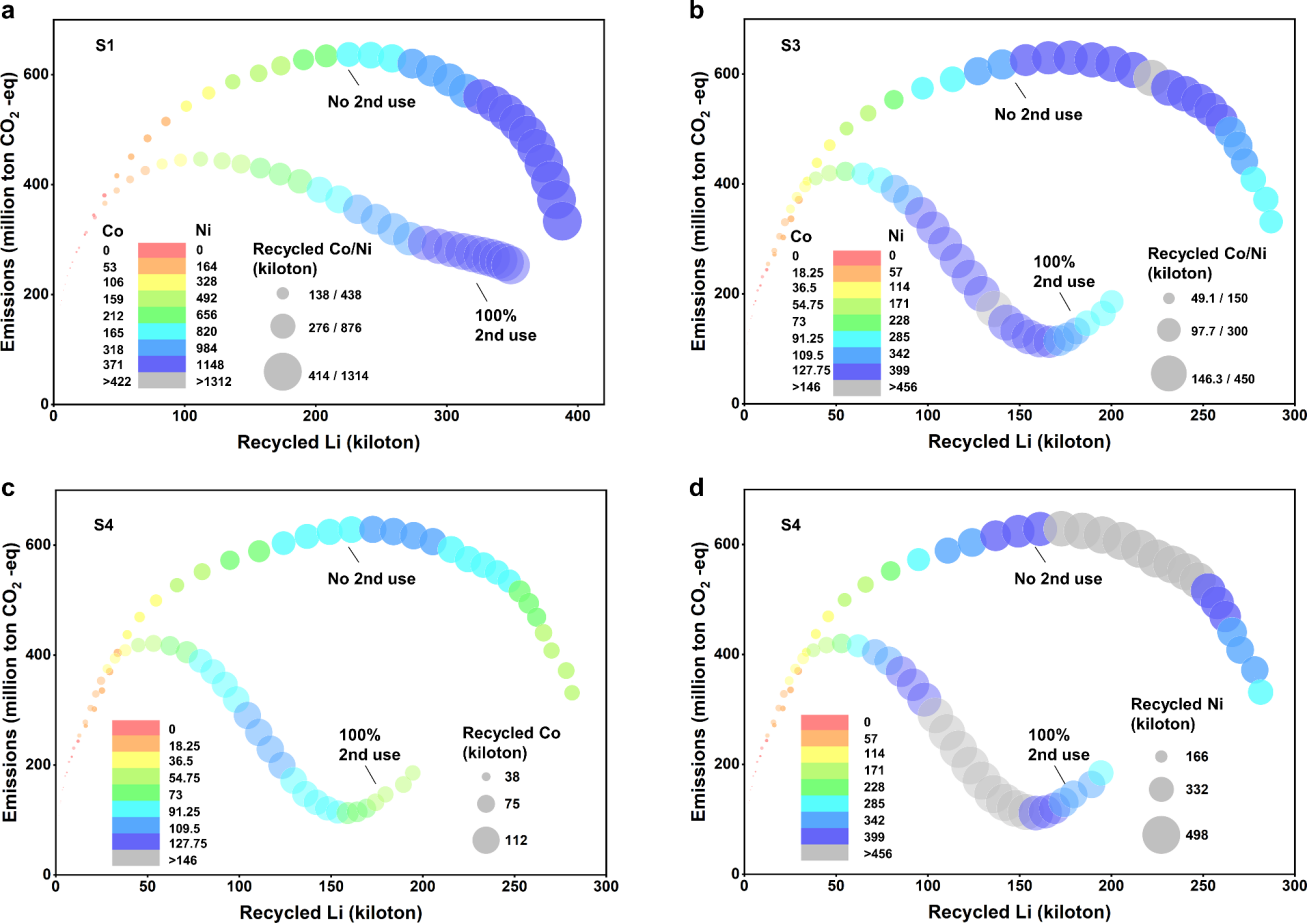
The amount of recycled cobalt and nickel and the life-cycle carbon emissions of EV batteries, relative to recycled lithium, in the studied system. (**a**) S1 and S2 (S1 and S2 share a similar pattern). (**b**) S3. (**c**) The amount of recycled cobalt and the life-cycle carbon emissions of EV batteries, relative to recycled lithium in S4. (**d**) The amount of recycled nickel and the life-cycle carbon emissions of EV batteries, relative to recycled lithium in S4. The DCR method is employed for demonstration, and all recycling methods exhibit a similar pattern. The color and size of the circles represent the amount of recycled cobalt and nickel, while the trend of the line illustrates the relationship between life-cycle carbon emissions and the amount of recycled lithium. The amount of recycled lithium increases over time.

The amount of recycled nickel and cobalt exhibits an almost linear relationship with that of recycled lithium under S1 and S2 (see Fig. 7a). However, under S3 and S4, the growth rate of recycled cobalt and nickel is slower than that of recycled lithium, attributed to the decreasing share of NCM. As the NCM share further diminishes, the recycling of cobalt and nickel declines, while the amount of recycled lithium increases, as reflected by the color and size of the circles (see Fig. 7b, c and d).

When second use is not implemented, life-cycle carbon emissions of the system increase with the quantity of recycled lithium at a gradually slowing rate before declining. This trend is observed across all scenarios (as shown by the lines in Fig. 7). This is primarily due to higher battery production and lower recycling in the early stage, followed by a significant increase in recycling and a decrease in new battery production in the later stage. However, with 100% adoption of second use, carbon emissions experience a rebound under S3 and S4 (see Fig. 7b, c and d), primarily due to the significant decrease in carbon emission reduction benefits from second use, driven by the greener electricity mix. It is evident that second use effectively reduces life-cycle carbon emissions, albeit at the cost of a sharp decrease in recycled materials. This pattern holds true across all recycling methods.

## Discussion

This study predicts that the stock of EVs in China will increase to 143 million by 2030 and 662 million by 2050, representing 13- and 62-fold increases from 2020 levels under the baseline scenario. Annual EV production in 2050 is projected to reach 86 million units, compared to 63.3 million units of Liu et al.^49^. The difference is primarily attributed to the higher EV penetration assumed in this study, reflecting the rapid increase in EV share in vehicle sales since 2022. Correspondingly, material demand for lithium, cobalt and nickel in 2030 is projected to be 137 kilotons, 168 kilotons, and 522 kilotons, respectively – 11-, 12-, and 12-times higher than 2020 levels. These figures closely align with the findings of Jones et al.^50^.

Our results demonstrate that adopting LFP-dominant pathways can significantly mitigate critical metal supply shortages for EVs. When EOL considerations are absent, this study shows that replacing NCMs with LFPs (S3) could avoid a minimum of 38.9 million tons (60.1%) of metal demand relative to the baseline. Additionally, at least 309.0 million tons (1.7%) of cumulative carbon emissions could be reduced. High-nickel strategies (S4) contribute to decreasing cobalt demand, albeit with an increased use of nickel. However, the impact on lithium demand is limited, and the overall demand for metals remains relatively unchanged. The carbon reduction effect is also modest, at 0.2% compared to S3. Considering the scarcity of cobalt and the abundance of nickel in global reserves^39^, opting for a high-nickel path^15^ appears more reasonable. However, considering China’s established cobalt production capability and the 20% increase in identified cobalt reserves from 2019 to 2023^39^, China is inclined to adhere to a medium-nickel strategy.

Our results also highlight the significant potential of battery recycling and remanufacturing in reducing raw metal use. Under LFP-dominant scenarios, recycling can satisfy demand for cobalt and nickel, contributing up to 80% to their use. However, a challenge arises as a minimum of 20% of lithium demand remains unanswered. Primary supply may need to more than double^13^, reaching 4,752 kilotons, to address this gap, which would account for 18.3% of the global lithium reserve. This, however, exceeds China’s total lithium reserve of 2,000 kilotons^39^. Regrettably, future battery technologies such as lithium-sulfur and all-solid-state batteries are poised to exacerbate the lithium supply shortage.

The carbon emission reduction potential of recycling without second use is limited, with average contributions of 3.4%, 2.8%, 2.1%, and 0.8% among the four scenarios of DCR-C, DCR-B, HR, and PR, respectively. The results contrast with those of micro-level LCA studies, which have emphasized the significant contribution of battery recycling^21,45^. This difference is mainly due to the relatively small proportion of carbon emissions from battery production compared to those from battery use within the broader system. Achieving 100% second use of retired LFPs boosts the maximum potential average carbon emission reduction to 37.9%, 37.5%, 37.1%, and 36.0%, respectively. LFP-dominant scenarios exhibit significantly higher carbon emission reduction potentials. However, drawbacks are apparent. Second use of retired LFPs notably widens the gap in lithium supply to over 40%. Determining the proportion of second use for retired LFPs should be approached cautiously. Additionally, considering the sharply reduced carbon reduction benefits of second use after 2050, it is recommended to significantly lower the proportion of second use at that time.

Considering the trade-offs between material supply and life-cycle carbon emissions, it is imperative to enhance the collection rate of retired EV batteries as much and as soon as possible. It should also be noted that we exclude the influence of stationary energy storage to avoid excessive complexity. Despite this limitation, our results remain valid, as the rate of secondary use does not impact total lithium demand when considering the energy storage battery system as a whole. If the lithium demand of the EV system alone cannot be satisfied, this shortfall would only be exacerbated by the inclusion of stationary energy storage.

## Conclusions

This study assesses the material demand and life-cycle carbon emissions of electrifying road transportation in China, with a focus on EOL strategies considering the market’s preference for LFP batteries. The findings reveal that adopting an LFP-dominant approach can significantly reduce critical material demand and carbon emissions, while recycling and reusing batteries further enhance these benefits. However, there is a trade-off between lithium supply shortages and carbon emission reduction, highlighting the conflict between ensuring lithium material security and achieving greater carbon emission reductions. The study also explores the lithium demand of emerging battery technologies, such as LSB and ASSB, only to find that they require more lithium.

This study has several limitations. First, data prior to 2021 are used to establish the baseline scenario. While this approach is appropriate for this study to investigate the impacts of transitioning from NCMs to LFPs, readers are advised to interpret the findings with caution. In addition, economic factors and general energy storage systems are excluded from the system boundary due to data limitations. Future studies are recommended to incorporate economic benefits and extend the system boundary when comparing different recycling methods and discussing the importance of second use and electricity storage by lithium batteries^51–53^. Furthermore, this study does not thoroughly consider the import and export effect for material security due to its complexity. Future studies are encouraged to explore and unveil its influence. Lastly, this study focuses solely on climate change impacts related to carbon emissions, excluding other environmental impacts. Given the potential for burden shifting in battery technology, future studies could broaden the scope to include additional environmental impacts.

## Electronic supplementary material

Below is the link to the electronic supplementary material.

Supplementary Material 1

## Data Availability

Data that support the findings of this study are provided in supplementary information and is also available through the corresponding author upon reasonable request.

## References

[1] Yu, F. et al. Techno-economic analysis of residential building heating strategies for cost-effective upgrades in European cities. *Iscience* **26** (2023). 10.1016/j.isci.2023.107541 PMC10480630 37680479

[2] IEA & Global *EV Outlook 2023* (International Energy Agency, 2023).

[3] Automobile Inspection & Registration Information Association. *Statistics on the number of vehicles owned*, (2023). https://www.airia.or.jp/publish/book/car.html

[4] CEIC. *South Korea Number of Registered Vehicles*, (2023). https://www.ceicdata.com/en/indicator/korea/number-of-registered-vehicles

[5] Chinese central government. *The number of nationla motor vehicles has reached 417 million and the number of drivers has exceeded 500 million*, (2023). https://www.gov.cn/xinwen/2023-01/11/content_5736278.htm

[6] Wang, H. et al. China’s electric vehicle and climate ambitions jeopardized by surging critical material prices. *Nat. Commun.* **14**, 1246 (2023). 36870994 10.1038/s41467-023-36957-4 PMC9985616

[7] Song, J. et al. Material flow analysis on critical raw materials of lithium-ion batteries in China. *J. Clean. Prod.* **215**, 570–581 (2019).

[8] Zhang, C., Zhao, X., Sacchi, R. & You, F. Trade-off between critical metal requirement and transportation decarbonization in automotive electrification. *Nat. Commun.* **14**, 1616. 10.1038/s41467-023-37373-4 (2023). 37041146 10.1038/s41467-023-37373-4 PMC10090058

[9] Castelvecchi, D. Electric cars and batteries: how will the world produce enough? *Nature* **596**, 336–339 (2021). 34404944 10.1038/d41586-021-02222-1

[10] Baars, J., Domenech, T., Bleischwitz, R., Melin, H. E. & Heidrich, O. Circular economy strategies for electric vehicle batteries reduce reliance on raw materials. *Nat. Sustain.* **4**, 71–79 (2021).

[11] Richter, J. L. A circular economy approach is needed for electric vehicles. *Nat. Electron.* **5**, 5–7 (2022).

[12] Xu, C. et al. Future material demand for automotive lithium-based batteries. *Commun. Mater.* **1**, 99 (2020).

[13] Zeng, A. et al. Battery technology and recycling alone will not save the electric mobility transition from future cobalt shortages. *Nat. Commun.* **13**, 1341 (2022). 35292628 10.1038/s41467-022-29022-z PMC8924274

[14] Harper, G. et al. Recycling lithium-ion batteries from electric vehicles. *Nature* **575**, 75–86 (2019). 31695206 10.1038/s41586-019-1682-5

[15] Li, W., Erickson, E. M. & Manthiram, A. High-nickel layered oxide cathodes for lithium-based automotive batteries. *Nat. Energy*. **5**, 26–34 (2020).

[16] Wang, M. et al. Recycling of lithium iron phosphate batteries: Status, technologies, challenges, and prospects. *Renew. Sustain. Energy Rev.* **163**10.1016/j.rser.2022.112515 (2022).

[17] Verma, S., Dwivedi, G. & Verma, P. Life cycle assessment of electric vehicles in comparison to combustion engine vehicles: A review. *Mater. Today: Proc.* **49**, 217–222 (2022).

[18] Shafique, M., Azam, A., Rafiq, M. & Luo, X. Life cycle assessment of electric vehicles and internal combustion engine vehicles: A case study of Hong Kong. *Res. Transp. Econ.* **91**, 101112 (2022).

[19] Hawkins, T. R., Singh, B., Majeau-Bettez, G. & Strømman, A. H. Comparative environmental life cycle assessment of conventional and electric vehicles. *J. Ind. Ecol.* **17**, 53–64 (2013).

[20] Xia, X. & Li, P. A review of the life cycle assessment of electric vehicles: Considering the influence of batteries. *Sci. Total Environ.* **814**, 152870 (2022). 34990672 10.1016/j.scitotenv.2021.152870

[21] Chen, Q. et al. Investigating carbon footprint and carbon reduction potential using a cradle-to-cradle LCA approach on lithium-ion batteries for electric vehicles in China. *J. Clean. Prod.* **369**, 133342 (2022).

[22] Lai, X. et al. Investigating greenhouse gas emissions and environmental impacts from the production of lithium-ion batteries in China. *J. Clean. Prod.* **372**, 133756 (2022).

[23] Xia, X., Li, P., Xia, Z., Wu, R. & Cheng, Y. Life cycle carbon footprint of electric vehicles in different countries: A review. *Sep. Purif. Technol.*, **122063** (2022).

[24] Shafique, M. & Luo, X. Environmental life cycle assessment of battery electric vehicles from the current and future energy mix perspective. *J. Environ. Manage.* **303**, 114050 (2022). 34872799 10.1016/j.jenvman.2021.114050

[25] Jiang, R., Wu, C., Song, Y. & Wu, P. Estimating carbon emissions from road use, maintenance and rehabilitation through a hybrid life cycle assessment approach–A case study. *J. Clean. Prod.* **277**, 123276 (2020).

[26] Plötz, P. Hydrogen technology is unlikely to play a major role in sustainable road transport. *Nat. Electron.* **5**, 8–10. 10.1038/s41928-021-00706-6 (2022).

[27] Hardman, S. & Tal, G. Understanding discontinuance among California’s electric vehicle owners. *Nat. Energy*. **6**, 538–545 (2021).

[28] Chen, Y. et al. Provincial and gridded population projection for China under shared socioeconomic pathways from 2010 to 2100. *Sci. Data*. **7**, 83 (2020). 32152299 10.1038/s41597-020-0421-y PMC7062824

[29] Dai, K., Shen, S. & Cheng, C. Evaluation and analysis of the projected population of China. *Sci. Rep.* **12**, 3644 (2022). 35256676 10.1038/s41598-022-07646-x PMC8901741

[30] Huo, H. & Wang, M. Modeling future vehicle sales and stock in China. *Energy Policy*. **43**, 17–29 (2012).

[31] Qiu, S., Lei, T., Wu, J. & Bi, S. Energy demand and supply planning of China through 2060. *Energy* **234**, 121193 (2021).

[32] Lu, Z. et al. Washington DC, United States,. in *Transportation research board 98th annual meeting* (2019).

[33] Guo, X., Zhang, J. & Tian, Q. Modeling the potential impact of future lithium recycling on lithium demand in China: A dynamic SFA approach. *Renew. Sustain. Energy Rev.* **137**10.1016/j.rser.2020.110461 (2021).

[34] Wu, Y., Yang, L., Tian, X., Li, Y. & Zuo, T. Temporal and spatial analysis for end-of-life power batteries from electric vehicles in China. *Resour. Conserv. Recycl.* **155**10.1016/j.resconrec.2019.104651 (2020).

[35] Chen, M. et al. Recycling end-of-life electric vehicle lithium-ion batteries. *Joule* **3**, 2622–2646 (2019).

[36] Hua, Y. et al. Sustainable value chain of retired lithium-ion batteries for electric vehicles. *J. Power Sources*. **478**, 228753 (2020).

[37] Plotz, P., Funke, S. A., Jochem, P. & Wietschel, M. CO(2) mitigation potential of plug-in hybrid electric vehicles larger than expected. *Sci. Rep.* **7**, 16493. 10.1038/s41598-017-16684-9 (2017). 29184118 10.1038/s41598-017-16684-9 PMC5705705

[38] Maisel, F., Neef, C., Marscheider-Weidemann, F. & Nissen, N. F. A forecast on future raw material demand and recycling potential of lithium-ion batteries in electric vehicles. *Resour. Conserv. Recycl.* **192**, 106920 (2023).

[39] U.S. Geological Survey. *Mineral Commodity Summaries 2023* (U.S. Geological Survey, 2023).

[40] Popien, J. L., Thies, C., Barke, A. & Spengler, T. S. Comparative sustainability assessment of lithium-ion, lithium-sulfur, and all-solid-state traction batteries. *Int. J. Life Cycle Assess.* **28**, 462–477. 10.1007/s11367-023-02134-4 (2023).

[41] Ahmadi, L., Young, S. B., Fowler, M., Fraser, R. A. & Achachlouei, M. A. A cascaded life cycle: Reuse of electric vehicle lithium-ion battery packs in energy storage systems. *Int. J. Life Cycle Assess.* **22**, 111–124 (2017).

[42] Zhu, X., Wang, S. & Wang, L. Life cycle analysis of greenhouse gas emissions of China’s power generation on spatial and temporal scale. *Energy Sci. Eng.* **10**, 1083–1095 (2022).

[43] Jiang, R. et al. Factors influencing the adoption of renewable energy in the US residential sector: an optimal parameters-based geographical detector approach. *Renew. Energy*. **201**, 450–461 (2022).

[44] IEA. *China’s Emissions Trading Scheme Designing Efficient Allowance Allocation* (International Energy Agency, 2020).

[45] Chen, Q. et al. Investigating the environmental impacts of different direct material recycling and battery remanufacturing technologies on two types of retired lithium-ion batteries from electric vehicles in China. *Sep. Purif. Technol.* **308**, 122966 (2023).

[46] López, A., Ramírez-Díaz, A., Castilla-Rodríguez, I., Gurriarán, J. & Mendez-Perez, J. Wind farm energy surplus storage solution with second-life vehicle batteries in isolated grids. *Energy Policy*. **173**, 113373 (2023).

[47] Gaines, L., Zhang, J., He, X., Bouchard, J. & Melin, H. E. Tracking flows of end-of-life battery materials and manufacturing scrap. *Batteries* **9**, 360 (2023).

[48] IEA. *The Role of Critical World Energy Outlook Special Report Minerals in Clean Energy Transitions* (International Energy Agency, 2022).

[49] Liu, B. et al. The impacts of critical metal shortage on China’s electric vehicle industry development and countermeasure policies. *Energy* **248**, 123646 (2022).

[50] Jones, B., Elliott, R. J. R. & Nguyen-Tien, V. The EV revolution: The road ahead for critical raw materials demand. *Appl. Energy*. **280**, 115072. 10.1016/j.apenergy.2020.115072 (2020). 33052165 10.1016/j.apenergy.2020.115072 PMC7545311

[51] Jiang, R., Wu, P. & Wu, C. Selecting the optimal network-level pavement maintenance budget scenario based on sustainable considerations. *Transp. Res. Part. D: Transp. Environ.* **97**, 102919 (2021).

[52] Wolinetz, M., Axsen, J., Peters, J. & Crawford, C. Simulating the value of electric-vehicle–grid integration using a behaviourally realistic model. *Nat. Energy*. **3**, 132–139 (2018).

[53] Powell, S., Cezar, G. V., Min, L., Azevedo, I. M. & Rajagopal, R. Charging infrastructure access and operation to reduce the grid impacts of deep electric vehicle adoption. *Nat. Energy*. **7**, 932–945 (2022).

