# Supplementary material for: Impact of electric vehicle battery recycling on reducing raw material demand and battery life-cycle carbon emissions in China
Source: Sci Rep. 2025 Jan 17;15:2267. doi: 10.1038/s41598-025-86250-1 (PMC11748696; doi:10.1038/s41598-025-86250-1)
Supplement: Supplementary file 1 — Supplementary Material 1 [file 41598_2025_86250_MOESM1_ESM.docx]

**Supplementary figures**


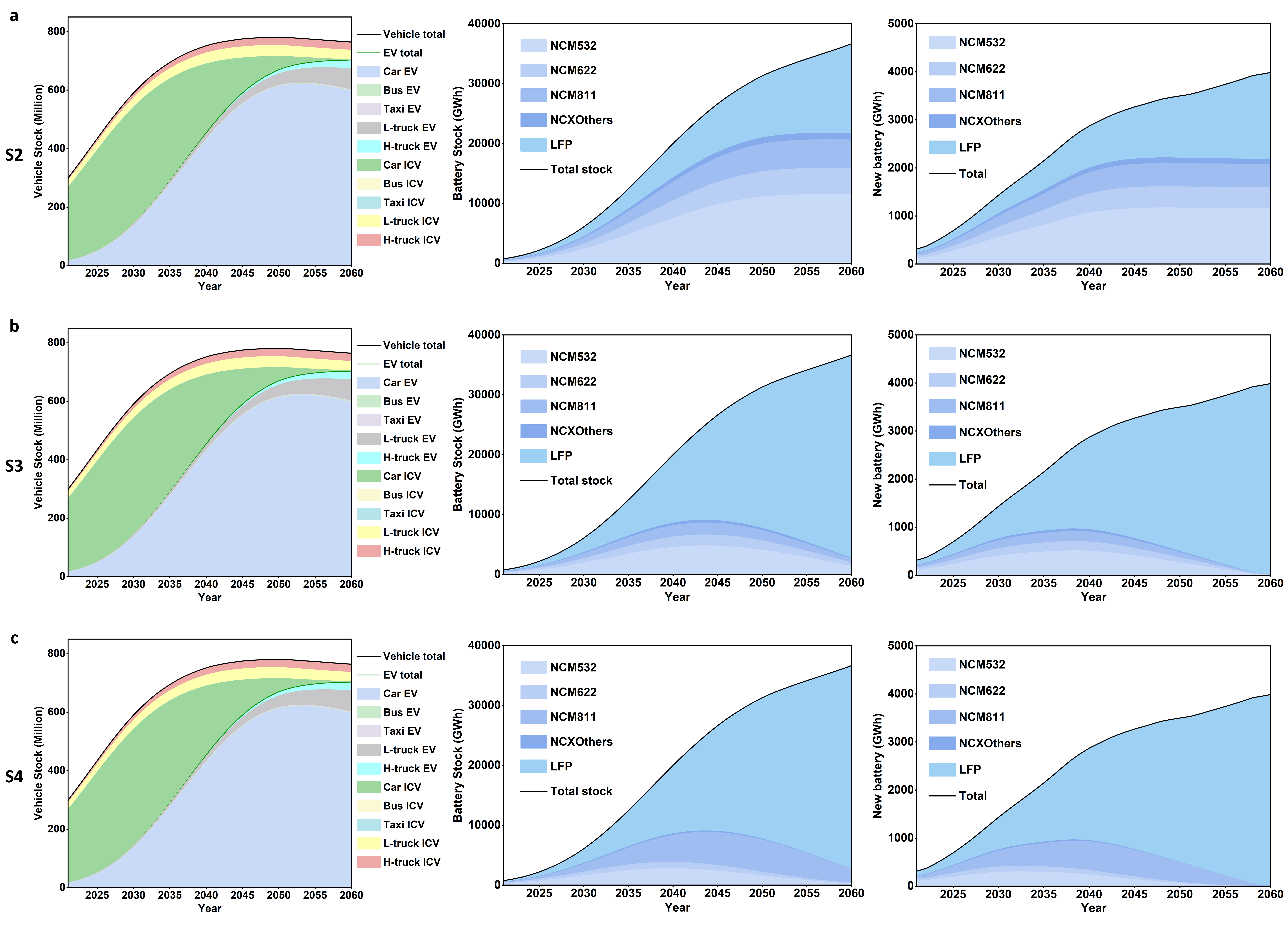


**Supplementary Fig 1. Battery stock, demand, and the corresponding metal demand. a** Results for scenario S2. **b** Results for scenario S3. **c** Results for scenario S4. S2 includes the extension of battery lifetime to ten years on top of the baseline scenario; S3 incorporates an LFP-dominant approach in addition to S2; S4 introduces high-nickel batteries on top of S3.

Electric vehicle (EV) stocks remain consistent across the three scenarios, slightly surpassing the baseline projection. By 2030, each scenario anticipates reaching 143.39 million units, with electric cars constituting 95% of this count. By 2060, the forecasts across scenarios show 702.97 million EVs, among which electric cars make up 85%. Battery stocks correspondingly total 6100.94 GWh in 2030 and grow to 36656.13 GWh in 2060. Interestingly, the annual demand for new batteries, expected to be about 11 times that of 2020 levels by 2030 and 34 times by 2060 across all scenarios, falls short compared to the baseline.


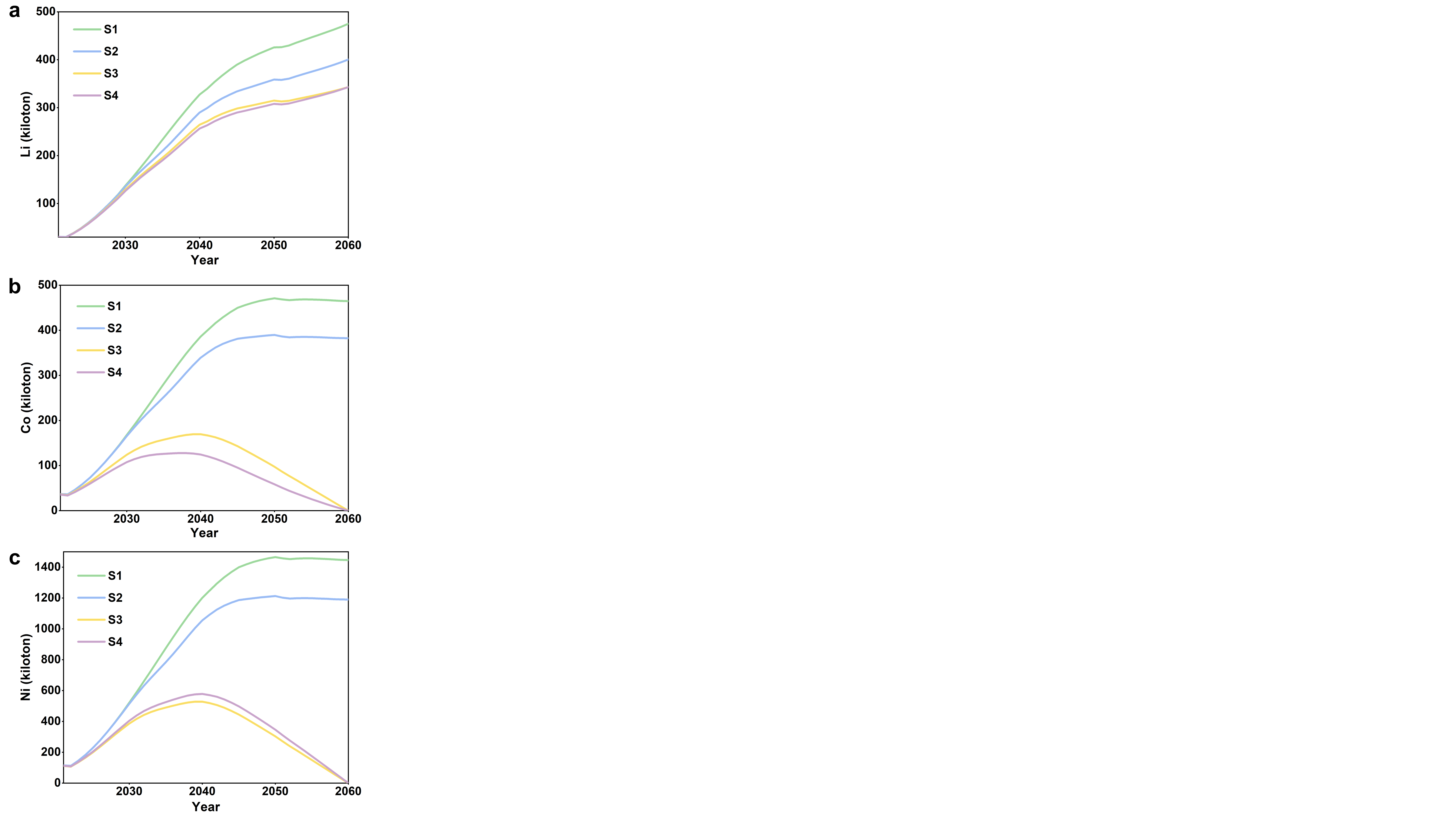


**Supplementary Fig 2**. **Annual metal demand. a** Lithium. **b** Cobalt. **c** Nickel. S1 – baseline scenario; S2 – S1 + extending battery lifetime to ten years; S3 – S2 + LFP-dominant approach; S4 – S3 + high-nickel batteries.

Supplementary Fig. 2 delineates the annual demand trends for lithium, cobalt, and nickel across various scenarios. In all four scenarios, lithium demand consistently ascends, maintaining an upward trajectory until 2060. The highest demand, recorded in the baseline scenario for 2060, reaches 475 kilotons. Contrarily, cobalt and nickel follow a divergent pattern: in scenarios S1 and S2, their demand continues to rise until around 2050, stabilizing thereafter. In scenarios S3 and S4, their peak demand occurs around 2040, notably lower than in S1 and S2, gradually diminishing to near-zero by 2060.


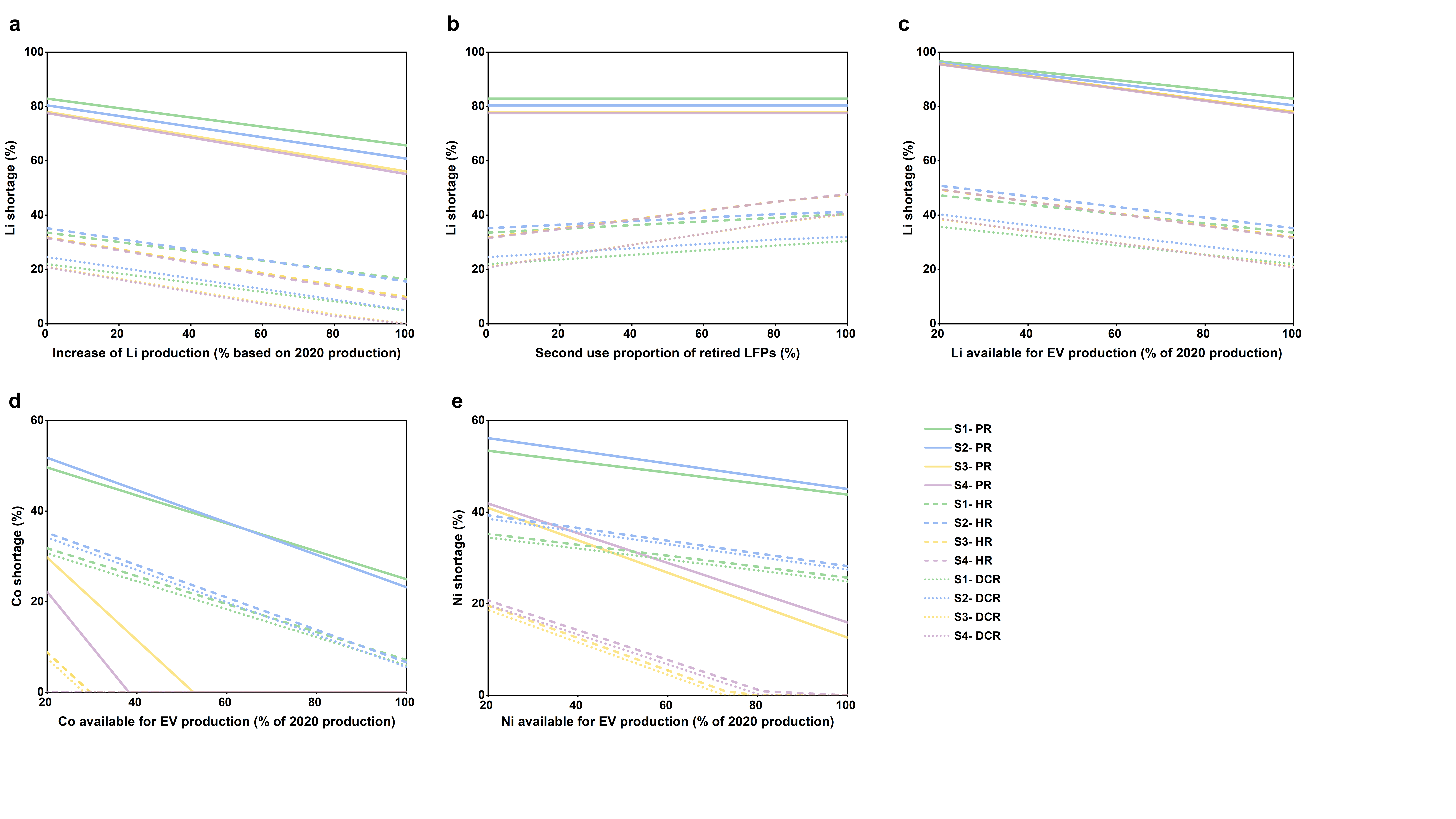


**Supplementary Fig 3**. **Sensitivity analysis results of metal shortages. a** Lithium shortage and increases in lithium production. **b** Lithium shortages and second-use proportion of retired LFPs. **c** Lithium shortage and available lithium for EV production. **d** Cobalt shortage and available cobalt for EV production. **e** nickel shortage and available nickel for EV production. PR - Pyrometallurgy Recycling; HR – Hydrometallurgical Recycling; DCR - Direct Cathode Recycling. S1 – baseline scenario; S2 – S1 + prolonging battery lifetime to ten years; S3 – S2 + LFP-dominant approach; S4 – S3 + high-nickel batteries.

Supplementary Fig. 3a illustrates the sensitivity of lithium shortages to increases in lithium production, assuming all lithium is available for EV battery production. The results indicate that lithium demand can only be satisfied under Scenarios 3 and 4 (S3 and S4) if the raw lithium supplied for EV battery production is at least double the total lithium produced in China in 2020. This assumes that all retired batteries are recycled using Direct Cathode Recycling (DCR) methods without a second-use phase. For Hydrometallurgical Recycling (HR) scenarios, the supply must be at least 240% of the total lithium produced in China in 2020. Additionally, S3 and S4 are highly sensitive to the proportion of retired Lithium Iron Phosphate (LFP) batteries reused and the availability of lithium for EV battery production, as demonstrated in Supplementary Figs. 3b and 3c.

In S3 and S4, cobalt demand can be met through any of the three recycling methods, whereas nickel demand can be addressed specifically by the HR or DCR approaches. This is contingent on the assumption that all produced metal is allocated for EV production. Supplementary Fig. 3e reveals that if the metal available for EV production falls below 81% of the levels produced in 2020, nickel demand can only be satisfied under S3 using DCR methods. Furthermore, if this proportion decreases to 72.8%, nickel demand becomes unmet. The supply of cobalt exhibits greater resilience; under S3, a cobalt shortfall only occurs when its supply for EVs dips below 28.2% of the 2020 production levels.


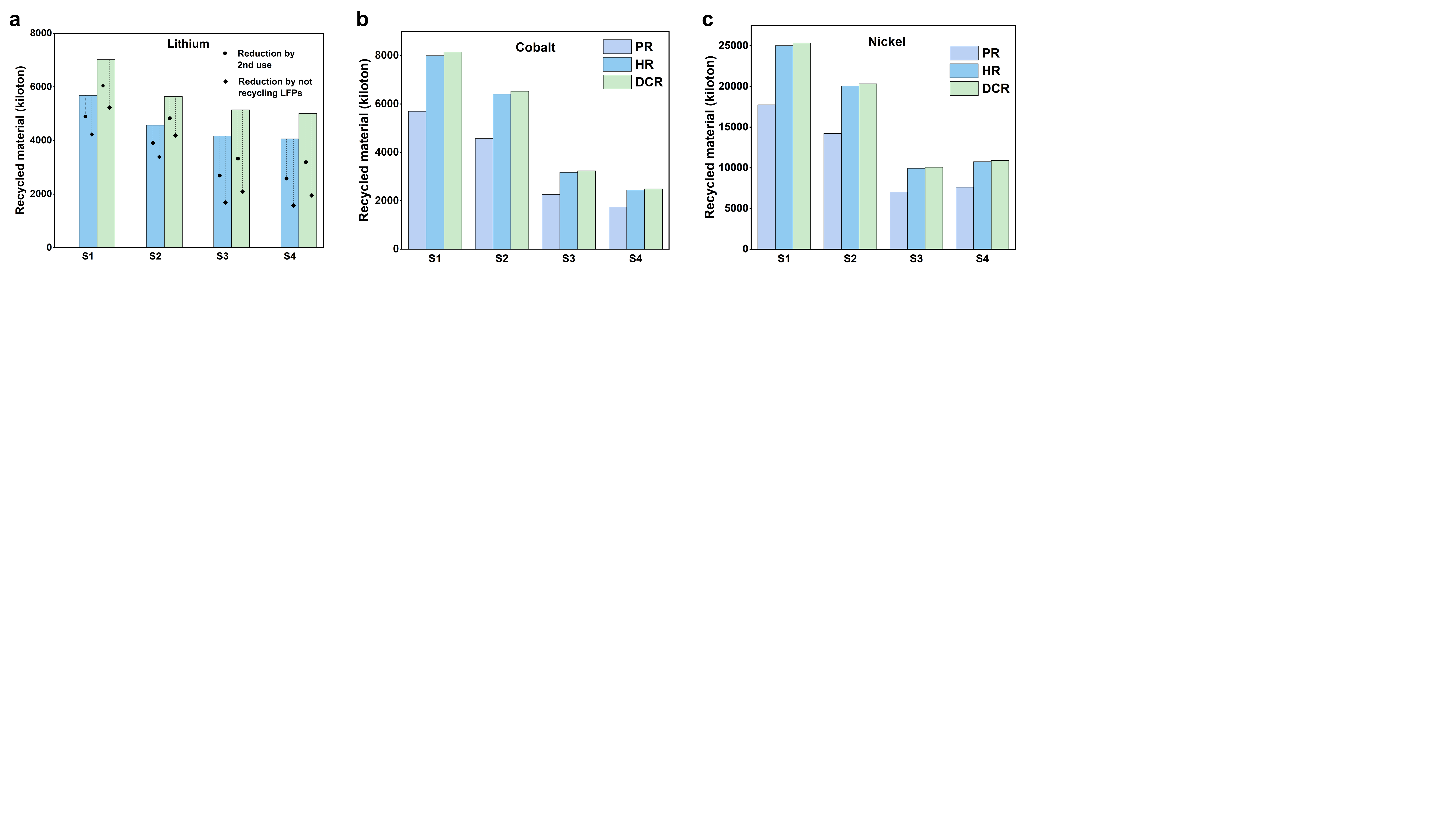


**Supplementary Fig 4**. **Cumulative recycled materials under various recycling methods and scenarios. a** Lithium. **b** Cobalt. **c** Nickel. PR - Pyrometallurgy Recycling; HR – Hydrometallurgical Recycling; DCR - Direct Cathode Recycling. S1 – baseline scenario; S2 – S1 + prolonging battery lifetime to ten years; S3 – S2 + LFP-dominant approach; S4 – S3 + high-nickel batteries.

In assessing recycling potential, Pyrometallurgy Recycling (PR) methods demonstrate the lowest efficacy, while DCR exhibits the highest, particularly notable for lithium. Within Scenario 1 (S1), the quantity of recycled lithium varies notably among methods: absent in PR, it amounts to 5,684.4 kilotons with HR, and increases to 7,017.78 kilotons with DCR. For cobalt and nickel, quantities recycled through these methods range from 5,702.21 to 8,146.01 kilotons and 17,750.21 to 25,357.45 kilotons, respectively. However, across the remaining three scenarios, the recycled metal quantities diminish in tandem with the reduction in annual new battery demand. Specifically, under Scenario 4 (S4), recycled lithium diminishes to 4,058.20 kilotons using HR and 5,010.12 kilotons with DCR. For cobalt under S4, quantities recycled through PR, HR, and DCR methods are 1,742.62 kilotons, 2,444.64 kilotons, and 2,489.45 kilotons, respectively. Correspondingly, nickel quantities recycled amount to 7,631.27 kilotons, 10,760.09 kilotons, and 10,901.81 kilotons using PR, HR, and DCR methods.

The second use of retired LFP batteries can significantly reduce the quantity of lithium requiring recycling, particularly in S3 and S4, which incorporate a high proportion of LFPs. Under these scenarios, such reduction amounts to 1,475.73 kilotons using HR methods and 1,821.89 kilotons with DCR. Furthermore, opting out of LFP recycling amplifies the reduction in recycled lithium quantities. This choice results in a decrease of 2,485.25 kilotons using HR and 3,067.97 kilotons employing DCR in S3 and S4, in comparison to complete recycling excluding cascade utilization.


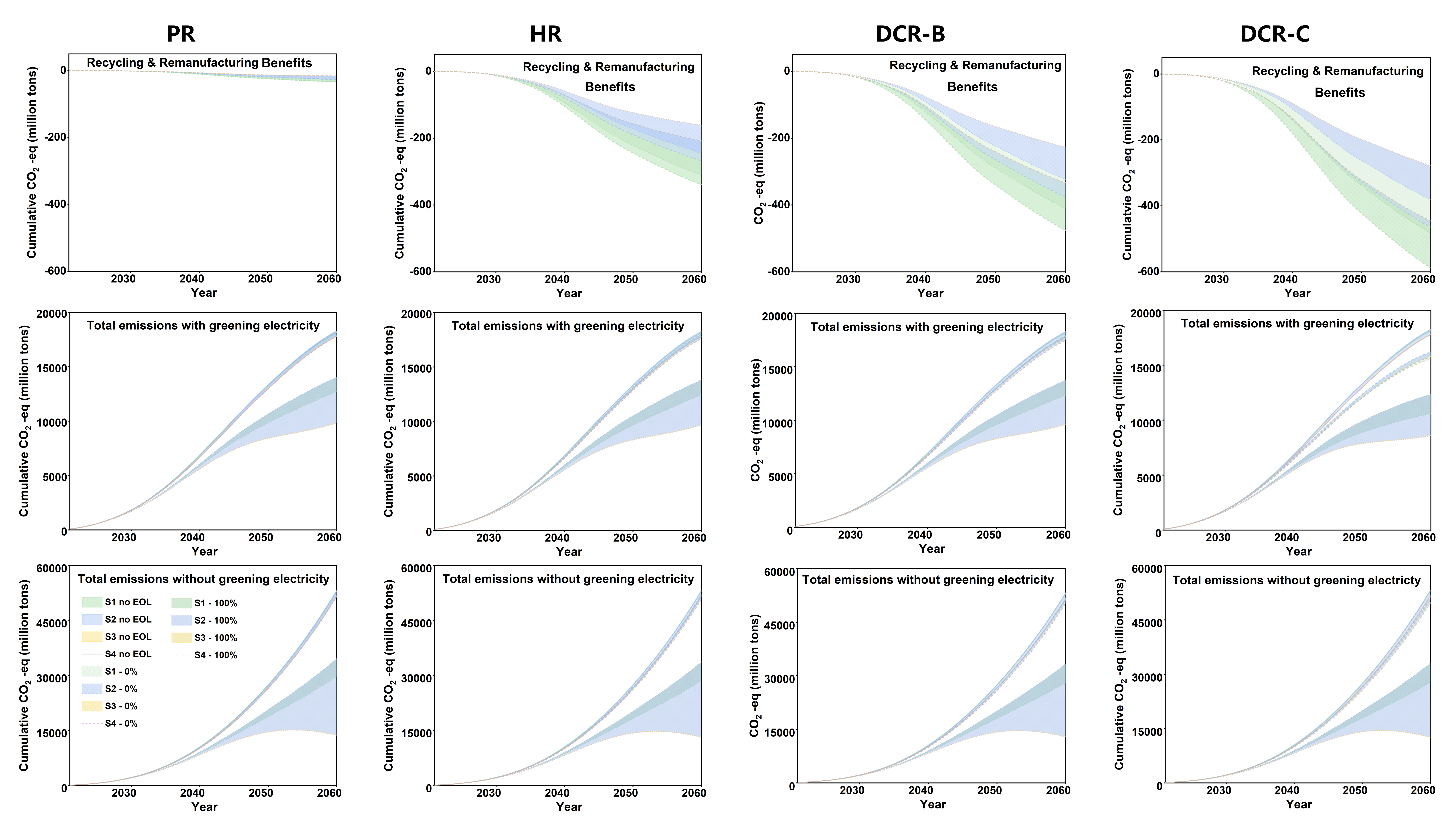


**Supplementary Fig 5**. **Cumulative life-cycle carbon emissions of the EV batteries in the studied system**. S1 – baseline scenario; S2 – S1 + prolonging battery lifetime to ten years; S3 – S2 + LFP-dominant path; S4 – S3 + high-nickel batteries. 0% and 100% represent scenarios where retired LFPs are subjected to 0% and 100% second use, respectively.

Supplementary Fig. 5 provides a comparative assessment of life-cycle carbon emissions across four distinct recycling methods. Among these approaches, DCR-C^1^ demonstrates the highest potential for emissions reduction, achieving a maximum cut of 588.13 million tons under the baseline scenario without second-use adoption. The full integration of second use decreases the maximum benefits of recycling and remanufacturing to 483.75 million tons. Nonetheless, the emissions reduction benefits derived from second use alone range from 4,215.54 to 7,965.49 million tons, varying across scenarios.

Notably, in scenarios considering or not considering the greening of the electricity mix, recycling retired batteries with 100% second use consistently offers significantly higher reductions compared to recycling without second use. Additionally, while S3 and S4 yield fewer benefits in recycling and remanufacturing, they exhibit the lowest total life-cycle carbon emissions among all scenarios, irrespective of recycling or second-use implementation. The impact of recycling LFPs appears negligible in these outcomes.

**Supplementary Tables**

**Supplementary Table 1. Configuration of key parameters across scenarios – EV penetration rate among new vehicles**^2,3^

| **Year** | **Car** | **Bus** | **Taxi** | **Light truck** | **Heavy truck** |
| --- | --- | --- | --- | --- | --- |
| 2020 | 10.5% | 75% | 59% | 4.5% | 0% |
| 2030 | 55% | 90% | 86.5% | 30% | 4.5% |
| 2040 | 95% | 100% | 100% | 50% | 30% |
| 2050 | 100% | 100% | 100% | 70% | 50% |
| 2060 | 100% | 100% | 100% | 85% | 70% |

**Supplementary Table 2. Configuration of key parameters across scenarios – battery ratio (baseline and scenario 2)^4^**

| **Year** | **Car & Taxi** | | **Bus** | | **Truck** | | **NCM811** | **NCM622** | **NCM532** | **Others** |
| --- | --- | --- | --- | --- | --- | --- | --- | --- | --- | --- |
|  | **LFP** | **NCM** | **LFP** | **NCM** | **LFP** | **NCM** |  |  |  |  |
| 2020 | 21.9% | 78.1% | 89.6% | 10.4% | 79.3% | 20.7% | 22% | 20% | 53% | 5% |
| 2030 | 21.9% | 78.1% | 89.6% | 10.4% | 79.3% | 20.7% | 22% | 20% | 53% | 5% |
| 2040 | 21.9% | 78.1% | 89.6% | 10.4% | 79.3% | 20.7% | 22% | 20% | 53% | 5% |
| 2050 | 21.9% | 78.1% | 89.6% | 10.4% | 79.3% | 20.7% | 22% | 20% | 53% | 5% |
| 2060 | 21.9% | 78.1% | 89.6% | 10.4% | 79.3% | 20.7% | 22% | 20% | 53% | 5% |

**Supplementary Table 3. Configuration of key parameters across scenarios – battery ratio (scenario 3)**

| **Year** | **Car & Taxi** | | **Bus** | | **Truck** | | **NCM811** | **NCM622** | **NCM532** | **Others** |
| --- | --- | --- | --- | --- | --- | --- | --- | --- | --- | --- |
|  | **LFP** | **NCM** | **LFP** | **NCM** | **LFP** | **NCM** |  |  |  |  |
| 2020 | 21.9% | 78.1% | 89.6% | 10.4% | 79.3% | 20.7% | 22% | 20% | 53% | 5% |
| 2030 | 41.4% | 58.6% | 92.2% | 7.8% | 84.5% | 15.5% | 22% | 20% | 53% | 5% |
| 2040 | 60.9% | 39.1% | 94.8% | 5.2% | 89.7% | 10.3% | 22% | 20% | 53% | 5% |
| 2050 | 80.5% | 19.5% | 97.4% | 2.6% | 94.8% | 5.2% | 22% | 20% | 53% | 5% |
| 2060 | 100% | 0% | 100% | 0% | 100% | 0% | 22% | 20% | 53% | 5% |

**Supplementary Table 4. Configuration of key parameters across scenarios – battery ratio (scenario 4)**

| **Year** | **Car & Taxi** | | **Bus** | | **Truck** | | **NCM811** | **NCM622** | **NCM532** | **Others** |
| --- | --- | --- | --- | --- | --- | --- | --- | --- | --- | --- |
|  | **LFP** | **NCM** | **LFP** | **NCM** | **LFP** | **NCM** |  |  |  |  |
| 2020 | 21.9% | 78.1% | 89.6% | 10.4% | 79.3% | 20.7% | 22% | 20% | 53% | 5% |
| 2030 | 41.4% | 58.6% | 92.2% | 7.8% | 84.5% | 15.5% | 41.5% | 15% | 39.8% | 3.7% |
| 2040 | 60.9% | 39.1% | 94.8% | 5.2% | 89.7% | 10.3% | 61% | 10% | 26.5% | 2.5% |
| 2050 | 80.5% | 19.5% | 97.4% | 2.6% | 94.8% | 5.2% | 80.5% | 5% | 13.3% | 1.3% |
| 2060 | 100% | 0% | 100% | 0% | 100% | 0% | 100% | 0% | 0% | 0% |

**Supplementary Table 5. Key assumptions for the four scenarios considered in this study.**

| **Scenarios** | **Descriptions** |
| --- | --- |
| **Scenario 1 (S1): baseline** | 8-year average EV battery lifetime;  Current market share: higher NCM market share (78.1%) than LFP;  Medium-nickel technologies dominating NCMs, with a composition of NCM532 at 53%, NCM622 at 20%, NCM811 at 22%, and Others at 5% |
| **Scenario 2 (S2): extending battery lifetime** | 10-year average EV battery lifetime;  Current market share: higher NCM market share (78.1%) than LFP;  Medium-nickel technologies dominating NCMs, with a composition of NCM532 at 53%, NCM622 at 20%, NCM811 at 22%, and Others at 5% |
| **Scenario 3 (S3): LFP-dominant** | 10-year average EV battery lifetime;  LFP gradually becoming the dominant technology in the Chinese EV battery market, with its market share increasing to 100% by 2060;  Medium-nickel technologies dominating NCMs, with a composition of NCM532 at 53%, NCM622 at 20%, NCM811 at 22%, and Others at 5% |
| **Scenario 4 (S4): high-nickel oriented** | 10-year average EV battery lifetime;  LFP gradually becoming the dominant technology in the Chinese EV battery market, with its market share increasing to 100% by 2060;  High-nickel technologies assuming dominance among NCMs over time, with the market share of NCM 811 gradually increasing to 100% by 2060 while others drop to 0% |

**Supplementary Table 6. Configuration of key parameters across scenarios – collection rate**

| **Year** | **Collection rate** |
| --- | --- |
| 2020 | 30% |
| 2025 | 48% |
| 2030 | 70% |
| 2035 | 80% |
| 2040 | 90% |
| 2045 | 90% |
| 2050 | 90% |
| 2055 | 90% |
| 2060 | 90% |

**Supplementary Table 7. Configuration of key parameters across scenarios – recycling rate^1^**

| **Recycling method** | **Collection rate** | | |
| --- | --- | --- | --- |
|  | **Lithium** | **Cobalt** | **Nickel** |
| Pyrometallurgical recycling (PR) | 0% | 70% | 70% |
| Hydrometallurgical recycling (HR) | 81.0% | 98.2% | 98.7% |
| Direct cathode recycling (DCR) | 100% | 100% | 100% |

Note: The DCR method was applied with additional material supplied to the cathode material during the recycling process; however, the supplied amount is negligible, and thus the recycling rate is considered 100%.

**Supplementary Table 8. Configuration of key parameters across scenarios – electricity mix**^5,6^

| **Year** | **Thermal power** | **Hydro** | **Wind** | **Photovoltaic** | **Nuclear** |
| --- | --- | --- | --- | --- | --- |
| 2020 | 67.9% | 17.0% | 6.0% | 3.5% | 5.6% |
| 2030 | 50.0% | 15.0% | 17.0% | 9.5% | 8.5% |
| 2040 | 32.0% | 12.0% | 28.0% | 15.5% | 12.5% |
| 2050 | 15.0% | 9.0% | 38.5% | 21.5% | 16.0% |
| 2060 | 0.0% | 15.0% | 38.5% | 35.2% | 11.3% |

**References**

1 Chen, Q. *et al.* Investigating the environmental impacts of different direct material recycling and battery remanufacturing technologies on two types of retired lithium-ion batteries from electric vehicles in China. *Separation and Purification Technology* **308**, 122966 (2023).

2 Hao, H. *et al.* Impact of transport electrification on critical metal sustainability with a focus on the heavy-duty segment. *Nature communications* **10**, 5398, doi:10.1038/s41467-019-13400-1 (2019).

3 Chinese central government. *Notice from the Ministry of Industry and Information Technology and seven other departments on organizing the pilot work of comprehensive electrification of public sector vehicles in advance areas.*, 2023).

4 Institute of Process Engineering, C. A. o. S. Report on China's Retired Electric Vehicle Battery Recycling Technology and Industry Development. (Institute of Process Engineering, Chinese Academy of Sciences. , Beijing, China., 2021).

5 Lai, X. *et al.* Investigating greenhouse gas emissions and environmental impacts from the production of lithium-ion batteries in China. *Journal of Cleaner Production* **372**, 133756 (2022).

6 Chen, Q. *et al.* Investigating carbon footprint and carbon reduction potential using a cradle-to-cradle LCA approach on lithium-ion batteries for electric vehicles in China. *Journal of Cleaner Production* **369**, 133342 (2022).
